# Antenatal couples’ counselling in Uganda (ACCU): study protocol for a randomised controlled feasibility trial

**DOI:** 10.1186/s40814-022-01049-5

**Published:** 2022-04-29

**Authors:** Vincent Mubangizi, Nuala McGrath, Jerome Kahuma Kabakyenga, Ingrid Muller, Beth L. Stuart, James P. Raftery, Sylvia Natukunda, Joseph Ngonzi, Clare Goodhart, Merlin Luke Willcox

**Affiliations:** 1 Mbarara University of Science and Technology, P.O. Box 1410, Mbarara, Uganda; 2 School of Primary Care, Population Sciences and Medical Education, Faculty of Medicine, University of Southampton, Southampton, UK; 3 Department of Social Statistics and Demography, Faculty of Social Sciences, University of Southampton, Southampton, UK; 4 Royal College of General Practioners, London, UK

**Keywords:** Antenatal, Film, Contraception, Family planning, Postpartum, Couples counselling, Birth preparedness, Birth planning, Uganda

## Abstract

**Background:**

Common avoidable factors leading to maternal, perinatal and neonatal deaths include lack of birth planning (and delivery in an inappropriate place) and unmet need for contraception. Progress has been slow because routine antenatal care has focused only on women. Yet, in Uganda, many women first want the approval of their husbands. The World Health Organization recommends postpartum family planning (PPFP) as a critical component of health care. The aim of this trial is to test the feasibility of recruiting and retaining participants in a trial of a complex community-based intervention to provide counselling to antenatal couples in Uganda.

**Methods:**

This is a two-group, non-blinded cluster-randomised controlled feasibility trial of a complex intervention. Primary health centres in Uganda will be randomised to receive the intervention or usual care provided by the Ministry of Health. The intervention consists of training village health teams to provide basic counselling to couples at home, encouraging men to accompany their wives to an antenatal clinic, and secondly of training health workers to provide information and counselling to couples at antenatal clinics, to facilitate shared decision-making on the most appropriate place of delivery, and postpartum contraception. We aim to recruit 2 health centres in each arm, each with 10 village health teams, each of whom will aim to recruit 35 pregnant women (a total of 700 women per arm). The village health teams will follow up and collect data on pregnant women in the community up to 12 months after delivery and will directly enter the data using the COSMOS software on a smartphone.

**Discussion:**

This intervention addresses two key avoidable factors in maternal, perinatal and neonatal deaths (lack of family planning and inappropriate place of delivery). Determining the acceptability and feasibility of antenatal couples’ counselling in this study will inform the design of a fully randomised controlled clinical trial. If this trial demonstrates the feasibility of recruitment and delivery, we will seek funding to conduct a fully powered trial of the complex intervention for improving uptake of birth planning and postpartum family planning in Uganda.

**Trial registration:**

Pan African Clinical Trials Registry PACTR202102794681952. Approved on 10 February 2021. ISRCTN Registry ISRCTN97229911. Registered on 23 September 2021

**Supplementary Information:**

The online version contains supplementary material available at 10.1186/s40814-022-01049-5.

## Background

Maternal mortality remains a major global concern, especially in sub-Saharan Africa where the maternal mortality ratio, although declining, is still high [1, 2]. Uganda’s maternal mortality ratio is estimated to be 336 maternal deaths per 100,000 live births [3]. The perinatal mortality rate is high at 38 per 1000 total births, and the under-five mortality rate is also still high at 64 deaths per 1000 live births [3]. High mortality rates are partly due to the lack of appropriate birth planning [4–9] and closely spaced pregnancies within the first year postpartum [9, 10].

Birth preparedness/complication readiness (BP/CR) is part of the package which should be provided under focused antenatal care in Uganda according to the Ministry of Health guidelines. Also, village health teams (VHTs) should provide BP/CR during health promotion and counselling in the community. A woman is classified as “well birth prepared” if she had accomplished at least three of the listed practices: identified skilled health professional, saved money and identified transport or had delivery kit/materials [5, 7, 11]. Studies from sub-Saharan Africa show that most women are not well prepared for birth and its complications [5, 11].

In Uganda, in 2016, 32% of sexually active unmarried women and 28% of currently married women had an unmet need for family planning whilst only 39% of married women were using a contraceptive method [3]. Unmet need was defined as “the proportion of women who (a) are not pregnant and not postpartum amenorrhoeic and are considered fecund and want to postpone their next birth for 2 or more years, or stop childbearing altogether, but are not using a contraceptive method; (b) have a mistimed or unwanted current pregnancy; or (c) are postpartum amenorrhoeic and their last birth in the last 2 years was mistimed or unwanted” [3].

A study done in Nepal, Senegal and Uganda showed that despite married women having visited a health facility in the past 12 months, the majority of them (87%, 74% and 40% in Nepal, Uganda and Senegal, respectively) still had an unmet need for family planning, and only a few of them (10%, 15% and 26% in Nepal, Senegal and Uganda, respectively) had discussed contraception during the last visit [12].

As in many other low-resource settings, women in Uganda are at high risk of unintended pregnancy soon after giving birth. The World Health Organization (WHO) recommends that contraceptive implants and intrauterine devices (IUDs) can safely be placed within 48 h of delivery [13]. Almost all postpartum women are medically eligible for long-acting reversible contraceptives (LARCs) [13], and LARCs should be offered as the first line given their low 1-year failure rates of 0.3% [14]. However, in spite of training health workers in Uganda on PPFP, uptake remains low [3]. Only 16% of women who delivered in health centres during their last birth in the 2 years preceding the survey received advice on family planning options before discharge [3]. There is a range of concerns about the use of contraceptives, like fear of side effects, many of which are derived from a lack of accurate understanding of contraception and a proliferation of myths about potential social and physical dangers associated with family planning [15–17]. These concerns need to be addressed.

Progress has been slow on improving birth planning and PPFP because routine antenatal care has focused only on women. Yet, the decisions about the timing and conditions of sexual relations, family size and access to health care including antenatal clinic (ANC) attendance and place of delivery are often made by men [18, 19], and most women first want approval of their husbands to use PPFP in Uganda [20]. In spite of efforts to encourage men to attend ANC with their partners in sub-Saharan Africa to increase opportunities for human immunodeficiency virus (HIV) testing [21], barriers remain to men attending antenatal clinics [21, 22].

When male partners are empowered with knowledge about ANC and engaged in birth preparedness, their participation in ANC increases, leading to an increase in the proportion of deliveries attended by a skilled birth attendant [23]. When a final decision on the location of birth [20] was jointly made by a couple, the woman was more likely to deliver under the assistance of skilled birth attendants (SBAs) compared to when the decision was individually made by either the husband or the wife alone [7]. A randomised controlled trial of prenatal counselling to women alone showed no impact on the uptake of contraception up to 12 months postpartum in Uganda [24]. Another randomised controlled study on antenatal counselling to women provided by health workers for PPFP in Tanzania showed an effect on contraceptive intentions, but not on use, reported at 6–15 months postpartum [25]. Three studies have shown that couples’ counselling can improve uptake of postpartum family planning: 3-h-long sessions in Egypt [26], one 20-min session in Pakistan [27] and repeated counselling sessions (every 2 months during the antenatal and postpartum periods; the average number of the visits and duration of sessions was not reported) delivered by community health workers in Bangladesh [28]. However, these contexts are very different from sub-Saharan Africa.

The intervention we will test in this trial has been developed on the basis of several qualitative studies involving communities and patients. A qualitative study in 2015 about barriers to the use of postpartum contraception [20], interviewing community members in south-west Uganda, suggested a need for better information and health education materials. Yet, written health education materials would not be widely accessible to the poor rural women, the majority of whom are unable to read a whole sentence [29]. Participants suggested the need for more awareness-raising on contraception and counselling of couples during antenatal clinics about postpartum contraception and to involve VHTs in providing basic information and awareness-raising [30]. This was after participants had watched a documentary and a drama focusing on the contraceptive implant developed using the person-based approach to intervention development [31] in south-west and central Uganda (Willcox et al., adapting the person-based approach to the development of health education films on family planning in Uganda, a low-income country, submitted).

VHTs are volunteer front-line community health workers who are recommended by the MoH for community education and health promotion for all diseases and have been used to deliver other health interventions in Uganda [32]. Trained health workers could then provide more specific counselling. These ideas have been extensively discussed with potential clients, VHTs and health workers, as well as district health teams and the Ministry of Health [30]. Training materials were adapted from those already approved by the Ministry of Health, Centre for Disease Control, USA, and the World Health Organization.

This study will generate evidence on how a complex intervention on antenatal couples’ counselling can be implemented and delivered in Uganda and how it is received in the local communities. It will also generate evidence on the possibility for VHTs to recruit and follow up pregnant women for up to 12 months after delivery, to collect key outcomes. We will also determine the approximate effect size and variability in the key outcomes. Data generated from the process evaluation will be crucial for optimising the local acceptability and chances of success of the couples’ counselling intervention, as well as for refining procedures for a fully powered trial to evaluate its effectiveness and cost-effectiveness. It is crucial to pilot a complex intervention, before conducting a full-scale trial as recommended by the Medical Research Council [33].

**Fig. 1:**
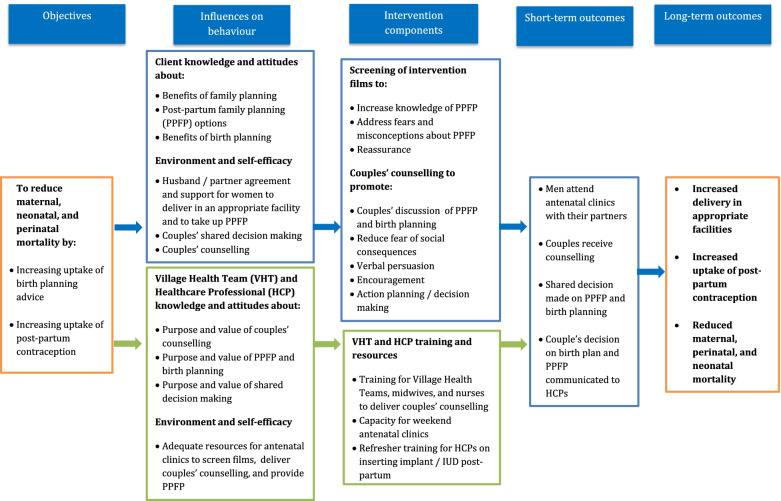
The logic model: antenatal couples’ counselling in Uganda on postpartum family planning

The logic model (Fig. 1) explains the overall objectives and expected short-term and long-term impacts and how we hope to achieve these by addressing influences on behaviour through the components of our intervention. The aim of this trial is to test the feasibility of recruiting and retaining participants in a trial of a complex community-based intervention to provide counselling to antenatal couples in Uganda. Our intervention addresses the issue of women needing the approval of their husbands, firstly by providing basic counselling to couples at home, encouraging men to accompany their wives to an antenatal clinic, and secondly by providing information and counselling to couples at antenatal clinics, to facilitate shared decision-making on the most appropriate place of delivery, and postpartum contraception. The intervention utilises the existing health system (both VHTs and health workers) to provide couples’ counselling to increase uptake of PPFP using antenatal care, delivery care and postnatal care services. The VHTs and health workers will be provided with training and mentoring to improve their counselling skills and empower them to deliver effective couples’ counselling.

The specific objectives are as follows:

- To assess the feasibility of recruiting pregnant women to a trial of the intervention, following them up and recording key outcomes, in both urban and rural areas
- To assess the feasibility of VHTs using smartphones to collect and record data on participants, using a secure data transfer system
- To measure the current levels of uptake of PPFP and estimate by how much this could be increased after couples’ counselling is introduced
- To assess the feasibility of combining couples’ counselling on family planning with counselling on birth planning in antenatal clinics in Uganda
- To evaluate the feasibility and acceptability of offering antenatal clinics and counselling at weekends
- To conduct a qualitative process evaluation aiming to identify operational reasons for failure or success in the implementation of the intervention and of the trial as a whole

## Methods

**Fig. 2:**
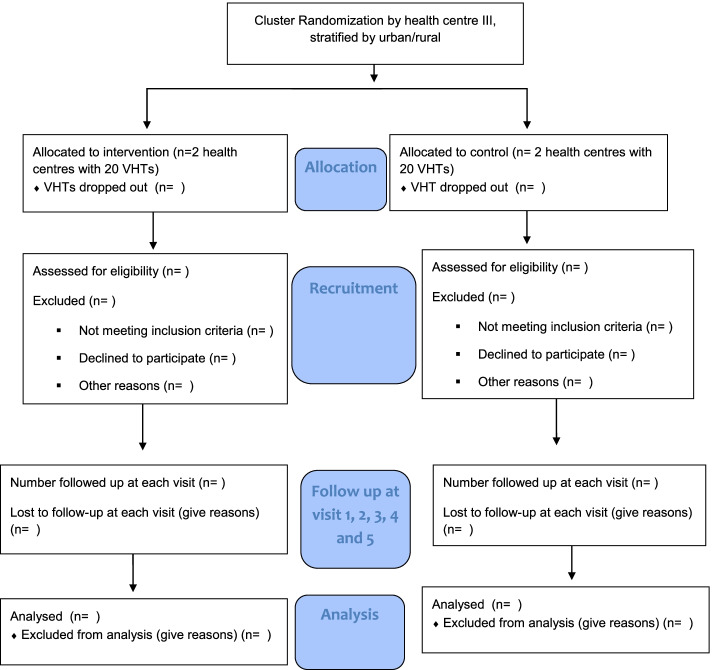
ACCU CONSORT 2010 flow diagram

A completed SPIRIT Checklist is available in Additional file 1. The CONSORT flow diagram for the study is shown in Fig. 2.

### Study design and setting

This is a feasibility non-blinded cluster-randomised controlled clinical trial of a complex intervention being conducted in primary health centres in Uganda. It is not possible to blind participants because the intervention is delivered through the local health centre. The use of cluster RCT design in this study is justified by the fact that the different components of the intervention package may not be delivered directly to individual participants but only applied at a cluster level [34, 35]. The unit for the administration of the intervention is a health centre rather than individuals. The cluster design minimises contamination by individuals to family, friends and community members since our intervention requires behavioural change as an outcome (uptake of birth planning and PPFP) [34, 35].

The unit “cluster” in this trial is a health centre III including the staff and patients in its catchment area. On average, the catchment area of a health centre III covers a population of 7846 women aged 15–49 years [36]. A health centre III cares for 4 to 6 parishes which are composed of 2–4 villages. The study area is in Mbarara District (rural) and Mbarara city (urban). According to the National Population and Housing Census (2014) Sub-County Report, women of reproductive age make up 25% (119,220/472,629) of the total population in the region [36].

### Eligibility criteria

This study targets pregnant women (and their partners) living in Mbarara in south-western Uganda. The primary participant will be the pregnant woman. If she meets the inclusion criteria, she will be included regardless of whether or not her partner consents to participate.

#### Inclusion criteria

Health centres to be included should meet the following criteria:

- Offering the continuum of antenatal care (ANC), delivery and postnatal care (PNC) services.
- Offering antenatal care services to more than 50 mothers per month.
- Have at least two midwives excluding those on the study and/or long-term sick leave.
- Providing modern contraceptive methods, including at least one long-term reversible method such as implants and/or intrauterine devices (IUDs).
- The facility must be connected to the national power grid so that they are able to show films.
- Health workers are willing to be involved in the study.
- VHTs in the area are functional and willing to be involved in the study.

Individual participants to be included should meet the following criteria:

- She is a resident of the area (in the catchment area of the relevant Health Centre III).
- The pregnancy is 7 months or less (self-reported).
- The woman plans to attend ANC and postnatal clinic (PNC) at the study health centre.
- The pregnant woman is in a relationship with the father of their expected child. It is not required that they live together but they must have been together for at least 6 months in a relationship, and see each other as their primary partner.
- Are 18 years old and above or they are emancipated minors able to consent.
- Informed consent is obtained.

#### Exclusion criteria

An individual will be excluded if she:

- Presents with severe medical/physical condition(s) making her unable to answer the questions at the time of the interview
- Has known causes of cognitive and functional impairment such as functional psychoses, depression and delirium, and alcoholism thus is not able to give informed consent

### Recruitment

In the catchment area of each of the four selected health facilities, all parishes will be selected for recruitment and follow-up of participants. In each parish, two VHTs will be invited to take part in the study (a total of 10 VHTs per area). The VHTs will be selected in a meeting organised at the health facility.

The participating VHTs will identify all pregnant women and their partners from their community registers of antenatal couples. VHTs will visit the pregnant women and their partners in their villages to screen them against the eligibility criteria, explain the study and request their consent to participate. Each screening will be recorded in a questionnaire on their smartphone using the COSMOS app, which will record the reason for exclusion of any woman who is not included. Eligible women will be asked for their written consent to participate (Additional file 2). If they consent, their partner will also be invited to consent, in particular, to answer the questions posed by the VHTs. If the partner does not consent to take part himself, the woman will still be included in the study (as an intention to treat analysis).

We will minimise selection bias by having a standardised data collection protocol, similar sources and methods of data collection in both intervention and control areas. The VHTs from both the intervention and control areas will be invited to attend a 2-day face-to-face training on the study, taking informed consent and data collection using the COSMOS app on a smartphone. Table 1 shows the training schedule.

**Table 1:** Training schedule for VHTs on taking informed consent and data collection

Time	Day 1	Day 2
8:30–9:00 am	Registration and welcome	Recap
9:00–9:30 am	Conducting a home visit	Review of study tool—2nd visit
9:30–10:00 am	Recruiting participants, explaining the study and obtaining consent	Use of the COSMOS app 2nd visit
10:00–10:30 am		Practice on the use of the COSMOS app 2nd visit
Health break		
11:00–11:30 am	Role-play to explain the study and obtain consent	Review of study tool—3rd visit
11:30 am–12:00 nn		Use of the COSMOS app 3rd visit
12:00–1:00 pm	Use of the COSMOS app, screening participants	Practice on the use of the COSMOS app 3rd visit
Lunch		
2:00–3:00 pm	Review of study tool—1st visit	Review of the study tool—4th and 5th visits—and use of the COSMOS app
3:00–4:00 pm	Use of the COSMOS app 1st visit	Practice on the use of the COSMOS app—4th and 5th visits
4:00–4:50 pm	Practice on the use of the COSMOS app—screening and first visit	ACCU study administration
4:50–5:00 pm	End of day evaluation	Closure

### Intervention

The complex intervention consists of the following components.

#### Training VHTs

In addition to the training provided to all VHTs (described above), in each intervention area, the VHTs will also be trained to provide basic information and counselling to antenatal couples in the community, encouraging them to attend a formal antenatal clinic, deliver in an appropriate place according to their level of risk, attend postnatal care clinic and use postpartum family planning. The information delivered by VHTs will also include birth preparation and complication awareness. The VHTs will take part in an additional face-to-face training course lasting 3 days, covering general counselling skills, couples’ counselling, identifying high-risk pregnancies, postpartum family planning, health education and gender-based violence (see Table 2). VHTs in the intervention group will have several opportunities to deliver counselling to the woman (with or without her partner) and to show her the health education films on family planning on their smartphone: at the baseline antenatal visit, at the second antenatal visit, and if still relevant at the first and second postpartum visits. They will encourage couples to attend antenatal clinics together at the intervention health facility, where a trained health worker will be able to provide further couples’ counselling. VHTs in the control group will not be able to show the films and will not have been trained to provide counselling but may still provide general health promotion advice according to the standard training that all VHTs receive.

**Table 2:** Training schedule for VHTs in the intervention

Time	Day 1	Day 2	Day 3
8:30–9:00 am	Registration and introductions	Recap	Recap
9:00–9:30 am	Pre-course questionnaire	Mediation skills	PPFP methods overview
9:30–10:00 am	Introduction to ACCU project and this course	Role-play on mediation skills	
10:00–10:30 am	General counselling skills	Solution-focussed model of couples’ counselling	Male involvement in family planning/RH services
Health break			
11:00 am–12:00 nn	REDI counselling framework	Role-plays on couples’ counselling	Health education and films show
12:00–1:00 pm	Role-plays on individual counselling	High-risk pregnancies Birth planning	Gender-based violence
Lunch			
2:00–3:00 pm	Couples’ counselling skills and self-awareness	Role-plays on birth planning and couples’ counselling	GBV role plays
3:00–4:00 pm	Forming alliances; directing communication	Postpartum family planning and concepts	General role-play practice
4:00–4:50 pm	Role-plays on couples’ counselling	Barriers to uptake of PPFP and common myths	Post-course questionnaire
4:50–5:00 pm	Daily evaluation		

*GBV* gender-based violence, *PPFP* postpartum family planning, *REDI* rapport building, exploration, decision-making, implementation, *RH* reproductive health

#### Training health workers

Health workers at the intervention health centre IIIs will be invited to attend a 6-day refresher training on general communication skills, couples’ counselling, information and practical skills for antenatal risk assessment, birth preparedness and postpartum contraception provision (see Table 3). Health workers will be asked to provide couples’ counselling to couples who come together to the antenatal clinic.

**Table 3:** Training schedule for health workers

Time	Day 1	Day 2	Day 3	Day 4	Day 5	Day 6
8:30–9:00 am	Registration and introductions	Recap	Recap	Recap	Recap	Recap
9:00–9:30 am	Pre-course questionnaire	High-risk pregnancies	Implant—insertion (including practice on models)	Participants practice insertion and removal of IUD on models	21: gender-based violence and role-plays	Clinical practicum
9:30–10:00 am	Introduction to the ACCU project and this course	Birth planning				
10:00–10:30 am	General counselling skills	Role-plays on birth planning and couples’ counselling	Implant—follow-up, management of side effects			
Health break						
11:00–11:30 am	The REDI counselling framework	Role plays on birth planning and couples’ counselling	Implant removal	Clinical practicum	Clinical practicum	21: records management
11:30 am 12:00 nn	Couples’ counselling skills and self-awareness exercise	Postpartum family planning and concepts	Practice implant removal on models			
12:00–1:00 pm		Barriers to uptake of PPFP	PPIUD assessment			Post-course questionnaire
Lunch						
2:00–3:00 pm	Role-play—practice	PPFP methods overview	Role-play—practice	Clinical practicum	Clinical practicum	Feedback on the course questionnaire
3:00–4:00 pm	Forming alliances and directing communication; 6: mediation skills	Implant—assessment and role-play	PPIUD insertion	Role-play practice	Feedback and discussion on lessons learnt from clinical practicum and challenges	Post-course evaluation ACCU study administration Closure
4:00–4:50 pm	Solution-focussed model of couples’ counselling	Health education and films	PPIUD follow-up, removal, post-insertion care			
4:50–5:00 pm	Daily evaluation					

#### Payment for health workers’ extra work

To compensate for providing couples’ counselling at weekends, the intervention health centres will receive 600,000 Ugandan shillings (about 120 UK pounds) monthly.

#### Screening of health education films in antenatal clinics and postnatal wards

Intervention health facilities will be provided with a TV screen and health education films about the contraceptive implant. They will be asked to screen the films daily. The films will also be shown in the waiting room of the antenatal clinic and of the postnatal clinic, so the couples will see them if and when they attend antenatal and postnatal clinics. The films have undergone an intensive development in Uganda using the person-based approach to intervention development [31] and have been approved by the Ministry of Health.

#### Supportive supervision visits

Supportive supervision visits will be undertaken at least every 2 months to VHTs and health facilities in the intervention arm, to ensure that the intervention is being delivered properly.

### Assignment of intervention or control

Clusters are at the level of the health centre III (sub-county). In all the villages surrounding a health facility, all the couples will be in the same group. Two urban and two rural health centre IIIs will be randomly selected from a list of all health centre IIIs in Mbarara District (rural) and Mbarara city (urban), in south-western Uganda. We will use a stratified randomisation according to urban/rural location. Participating health facilities will be randomised during a meeting in which the district health officer is invited to draw pieces of paper out of a hat, to determine whether each health centre is assigned to the intervention or control arm—so that the randomisation process is open and understood by all. It will not be possible to blind participants or care providers. If two of the selected sub-counties share a boundary, we will only include women intending to attend ANC and PNC in their area, in order to minimise contamination.

### Data collection and follow-up

All trial procedures are shown in the SPIRIT figure (Table 4). The questionnaires will be completed using the COSMOS digital data entry platform on smartphones (https://cosmos.tetratech.com/). This allows data entry even in the absence of a mobile phone network and upload of data at a later time into the COSMOS database. All participating VHTs will be provided with a smartphone which will remain the property of the research team until the end of the study, when the VHTs will be allowed to keep them. VHTs will pilot the use of smartphones for data entry using a training version of the COSMOS app for 2–3 weeks before starting recruitment. VHTs will be given paper case record forms to use as a backup if there are technical problems which prevent data entry on the phones. The research assistant would then enter data from paper case report forms into the electronic database. VHTs will be trained to store paper forms securely until they can be passed on to the research team.

**Table 4:** ACCU SPIRIT figure showing the schedule of enrolment, interventions and assessments

Time point	Study period					
	Screening	Post-allocation				Close-out
	***Screening visit***	***Antenatal 1 (baseline)***	***Antenatal 2 (after 1 month)***	***Postnatal 1 (1 week after delivery)***	***Postnatal 2 (6 months after delivery)***	***Postnatal 3 (12 months after delivery)***
**Enrolment**						
**Eligibility screen**	X					
**Informed consent**	X					
**Interventions**						
***Counselling on birth planning***		X	X			
***Counselling on PPFP***		X	X	X	X	
**Assessments**						
***Relevant demographics, medical/obstetric history***		X				
***Plans for birth and PPFP***		X	X			
***Use of antenatal clinic, delivery outcomes***				X		
***Use of PPFP***				X	X	X
***Maternal and child survival outcomes***				X	X	X

### Baseline antenatal visit

After a woman has consented to take part, the VHT may continue immediately with the baseline visit or may arrange to return at a later time. However, the baseline visit should be conducted no later than 7 months of pregnancy, to allow time for the couple to engage in counselling and decision-making before the delivery. The VHT will take the woman (and her partner, if he is present) through the baseline questionnaire (Additional file 3) about her demographic characteristics, any previous pregnancies, the current pregnancy and her future intentions regarding the place of birth and postpartum family planning.

### Follow-up visits

Village health teams in all areas will attempt to follow up on pregnant women and their partners a total of four times. VHTs will perform one antenatal follow-up visit at least 1 month after the baseline (data collection about use of antenatal clinic and intentions to use birth planning and PPFP). The postnatal visits will take place at about 1 week, 6 months and 1 year after giving birth (to collect data on place of delivery, uptake of postpartum contraception, perinatal and neonatal mortality). On each occasion, they will enter data into the relevant electronic questionnaire on their smartphone. VHTs in the intervention group will deliver additional counselling and will encourage couples to attend the health facility for couples’ counselling if needed, for couples who have not yet taken up postpartum contraception.

In each health facility, health workers will be asked to report aggregate data every month on the number of women attending ANC alone or with their partners, the number of couples counselled about PPFP, the number of deliveries and the number of women provided with PPFP. All participating health centres receive a payment of 100,000 Ugandan shillings per month (about 20 UK pounds) for their help with data collection.

### Adverse event reporting

In Uganda, domestic violence is common. According to the 2016 Uganda Demographic Health Survey [37], 56% of ever-married women and 44% of ever-married men have experienced physical, sexual or emotional violence by their current or most recent spouse/partner, and one in five have experienced physical violence in the preceding year. Therefore, it is likely that some of our participants will experience this during the course of the study, although we hope that our intervention may actually reduce the risk by fostering good communication and mutual understanding between partners in a couple. Nevertheless, at follow-up visits, the VHT will always ask briefly to speak separately with each member of the couple and will then ask screening questions about domestic violence, only if it is possible to speak in private.

If VHTs become aware of domestic abuse occurring within one of the included couples, they will counsel and facilitate the victim to report the case within existing structures—initially, this would be the focal person for gender-based violence at the health facility, or the Local Council I leader who is responsible for addressing these issues and offering support as appropriate, and/or the community development officer or police. If the victim does not feel ready, the VHT will follow up and counsel her/him, with the goal of supporting her/him to disclose through the appropriate channels. The research team will monitor and report the progress of that referral as part of adverse event reporting, including a report of whether on review it is associated with being in the study or not. Any serious adverse event (resulting in hospitalisation or death) will be reported to the Mbarara University Research Ethics Committee (MUREC) using their standard serious adverse event reporting form.

### Monitoring data quality

Data entry directly into the COSMOS system minimises the risk of data loss. We will control the quality of quantitative data collection using internal controls on the COSMOS system—for example, by checking the global positioning system (GPS) location of the interview and the time taken for the interview. We will also conduct spot checks—a researcher will repeat data collection for a random sample of about 5% of participants from each village health team member to ensure that the data has been captured accurately—if significant discrepancies are found, a larger sample will be repeated for that VHT. In the electronic database, we will use standardised data capture with drop-down boxes and data entry validation. Regular debriefing sessions will be held with VHTs for review of experience and discussion of difficulties.

### Process evaluation

In complex intervention trials, it cannot be assumed that the delivery of an intervention in “real life” will be exactly as planned in the design stage of a trial. Process evaluation can lead to a greater understanding of what works or does not and provide meaningful interpretation of the effects of an intervention to inform future implementation [33, 38]. During the intervention, we will conduct a qualitative process evaluation aiming to identify operational reasons for failure or success in the implementation of the intervention and of the trial as a whole. We will assess the intervention fidelity and experiences of the intervention (in the intervention group) through ongoing monitoring and supportive supervision visits.

Qualitative data will be collected using an interview guide to capture changes in implementation or contextual factors. The interview schedules will be altered in response to emerging themes developing from analysis which will take place concurrently with data collection. This will ensure that emerging themes from earlier data can be investigated in later data collections. We will hold the interviews and focus groups at least 3 months and up to 12 months after the delivery. We will start interviews and focus group discussions at least 6 months after the start of the trial and intervention. Interviews will be conducted by a member of the research team with training and experience in qualitative research, who is fluent in the local language, Runyankole. The interviews will take place in a location such as the participant’s home or private room at a health centre, guaranteeing privacy. They will be audio-recorded and will be transcribed verbatim and translated into English where English was not the language used during the interview, omitting any names so that transcripts are anonymous. Field notes will be taken to record non-verbal communication.

### Outcomes

#### Primary process/feasibility outcomes

The following will be the primary feasibility outcomes:

1. Percentage of eligible women who consent to participate and are recruited
2. Percentage of included women who:
  - Can be followed up and provide outcome data on place of delivery and uptake of family planning: to 1 week postpartum, to 6 months postpartum and to 1 year postpartum.
  - Whose partners agreed to participate in the project
  - Received individual counselling from the VHT: on one or more occasions and at each study visit
  - Received couples counselling from the VHT on one or more occasions
  - Attended the antenatal clinic at the health centre one or more times
  - Attended the antenatal clinic at the health centre with their partner one or more times
  - Received individual counselling at the antenatal clinic on PPFP
  - Received individual counselling at the antenatal clinic on birth planning
  - Received couples’ counselling at the antenatal clinic on PPFP
  - Received couples’ counselling at the antenatal clinic on birth planning
  - Were given correct advice on the recommended place of delivery (according to their level of risk)
  - Watched the health education films: with the VHT and at the antenatal clinic
3. Percentage of included couples who:
  - Were offered and attended antenatal clinics and counselling at weekends
  - Were counselled and consented for long-acting reversible methods of PPFP
4. Proportion of women/couples attending antenatal clinic who were offered counselling on both birth planning and use of long-acting reversible methods of PPFP at the same visit
5. Proportion of women/couples counselled who agreed on PPFP in a single session and proportion of women/couples counselled who agreed after attending extra sessions
6. Percentage of working days when the films on family planning are screened in antenatal clinics (in intervention areas)
7. Percentage of VHT visits when:
  - Counselling was delivered to the woman/couple about birth planning/family planning
  - Data was correctly entered directly into the COSMOS app on the smartphone
8. Costs of intervention/control—health economics

#### Secondary outcomes of the study

The secondary outcomes (estimates of effect size for the main trial) will be the following:

1. Percentage of included women who:
  - Delivered in an appropriate place, as recommended by the health worker according to their level of risk
  - Died (maternal death)
  - Suffered serious maternal morbidity
  - Took up postpartum family planning: LARC vs other methods and within 48 h/by 6 weeks/by 6 months/by 12 months
2. Percentage of women who initiated PPFP who are still using it: at 6 months and at 12 months
3. Percentage of babies born to included women/couples who:
  - Were live born vs stillborn (fresh/macerated)
  - Died within 7 days, 28 days or 12 months of birth
  - Had a serious illness (for example requiring hospital admission/causing long-term complications) within 7 days, 28 days or 12 months of birth
  - Lost a sibling during the 12-month follow-up period

### Sample size

There are four clusters—two intervention and two control areas—in the feasibility trial. In each of these areas, we will engage 10 VHTs to recruit participants. Each VHT will aim to recruit 35 women over a 6-month period, so in total, we will aim to recruit about 1400 women. A formal sample size calculation is not required for a feasibility study, but a sample of this size will allow us to estimate the key parameters for a future full-scale trial, including the variability, clustering and retention rate.

For the process evaluation, we will use a “purposive” sampling approach to select respondents (according to socio-demographic and organisational factors expected to influence the delivery or effectiveness of the intervention). We plan six separate FGDs, each of six to ten participants, two each for the following categories of individuals: (a) postpartum women—the age will determine their allocation to subgroups: adolescent girls (≤ 20 years), women 21 years and above; (b) men who are partners or husbands of women who deliver during the study; and (c) VHTs involved in the study. We will conduct about 20–30 in-depth interviews with clinic managers, health service providers, VHTs and couples. We will interview postpartum women and their partners, who took up, and who did not take up, the offer of couples’ counselling at home and at ANC and the offer of a contraceptive method, in order to understand the factors influencing their decisions, their experience of the intervention and their suggestions for improving it. We will also ask about the degree of contamination between study areas and will assess this as a feasibility outcome.

### Statistical analysis plan

The primary analysis will determine whether the study is feasible. Data will be explored descriptively and graphically for all feasibility outcomes including the intervention uptake, adherence, attrition, retention and the number of participants recruited per site. We will estimate the variance of proposed outcome measures; alongside previous literature, this will be helpful in a future sample size calculation. We will also estimate the intra-cluster correlation coefficient (ICC), and its 95% confidence interval, at both the randomisation and VHT level. Whilst these estimates are not by themselves sufficient for a future calculation, they can be compared to the existing literature and give some reasonable bounds for future estimates as well as allowing us to explore at which level clustering may be present so that this may be factored into the future design.

All participant data will be included in the analyses, including those who have withdrawn, unless the participant specifically requests that their data be removed. The pattern and frequency of missing data will be explored descriptively to determine whether there are outcomes or items on instruments that participants opted not to complete. This may inform the decision about measures for a full trial.

### Qualitative analysis plan

In-depth interviews and focus group discussions will be transcribed verbatim and translated into English, where English was not the language used during the interview, into Microsoft Word documents and analysed in Atlas.ti®. Thematic content analysis will be used [39]. Individual interviews or focus group discussions will constitute the units of analysis. At least two investigators will code the first three interviews/focus groups and will independently propose a coding framework. The coding framework will be discussed with the wider team before being finalised and applied to the interviews.

### Cost effectiveness

Quantitative data on the role of costs both formal and informal in relation to the intervention will help in the design of a cost-effectiveness study. The cost-effectiveness of the intervention versus control depends on the additional cost of the intervention plus any knock-on effects on other costs. In this feasibility stage, both of these will be explored. We will evaluate the different ways of delivering the intervention in relation to its cost, given that the method of delivery may have to vary by place. The standard questionnaires will include questions about the costs of different components of care in both groups. The aim will be to assess in the process evaluation the likely cost of the intervention if delivered in different ways and to guide how it might be delivered most efficiently in the main trial. The knock-on effects of such an intervention, which may be diffuse (such as travel to the venue, time off work and cost of measures to encourage spouse attendance) will also be explored qualitatively in the process evaluation. We will also explore in an open-ended way what other non-healthcare costs might be incurred by trial participants. One perspective often adopted for costing is that of the health care provider, and this will be a perspective included in the main trial.

Cost per birth averted is likely to be the measure of cost-effectiveness in the main trial but the appropriateness of this will also be explored particularly with the local health service leaders and taking account of other relevant work in the field and in terms of policy alternatives. Cost per quality-adjusted life year (QALY) is not usually seen as appropriate for interventions to do with fertility control. Thus, consideration will be given to the inclusion of other secondary outcomes by use of an impact inventory which will list all the effects, both to do with outcomes and resource use, of the intervention. The eventual cost-effectiveness analysis will compare the results from the trial with those in the relevant literature.

### Data management plan

Each participant will be given a unique identification number. Data entered via the COSMOS database is automatically backed up to 3 different data centre regions. All collected data is automatically deleted from mobile devices after successful synchronisation to the COSMOS database. Downloaded data and manually entered data will be stored on password-protected computers. All access to data is very tightly and strictly controlled by the University of Southampton. Transcripts from interviews and focus groups will also be anonymised and shared between the research groups. At the time of publication, we will make the anonymised data freely available on a data repository of the University of Southampton.

### Data security and confidentiality

COSMOS uses built-in advanced encryption technology (AES256) to safeguard data and meet obligatory commitments towards data protection. Data entry directly into the COSMOS system minimises the risk of data loss or breach of confidentiality. The VHTs will use a lockable cupboard to keep documents. The research assistant will monitor this and will collect study documents on a regular basis and transfer the documents to the secure locked cabinet in the study office. The study database will be password-protected and will be stored on a password-protected computer. Data entered into the study database will be anonymised.

Qualitative data will be recorded on a digital voice recorder, which will also be stored securely in a locked cabinet in the research office. The recordings will be transferred to a secure password-protected computer. They will be transcribed and translated into a Word document on the same computer where the recordings are downloaded, omitting any names so that transcripts are also anonymous. Once the transcripts have been checked and corrected, if necessary, the voice recordings will be deleted.

### Ethical and regulatory aspects

Randomised controlled trials have special ethical concerns and the ethical reason for randomly assigning the intervention arm is because we expect both arms to be at a state of equipoise. For the purpose of this feasibility trial, the research participants in the control arm have access to standard care that is deemed adequate healthcare by the Ugandan Ministry of Health.

The study was approved by MUREC (the Mbarara University of Science and Technology Research Ethics Committee, Ref #. 16/06-20) and the University of Southampton, Faculty of Medicine Ethics Committee (Ref #. ERGO 54459.R3). All the study methods and procedures will be conducted in accordance with MUREC’s guidelines and regulations. Written informed consent will be obtained from participants of the study. In the case of illiterate participants, they will be asked to use a thumbprint, and an independent witness will sign to confirm that they gave their consent freely in line with the MUREC guidelines. We will train all research assistants and village health teams in relevant principles of good clinical practice, particularly taking informed consent, and confidentiality. All protocol modifications will be communicated to MUREC for approval before they are implemented, and protocol modifications relevant to health care provision will be communicated to study sites. The currently approved version of the protocol is version 2, dated July 29, 2020. We are required to report any deviations from the protocol to the Mbarara University of Science and Technology Research ethics committee. The study will comply with the data protection guidelines of the University of Southampton.

This trial has an independent Trial Steering Committee (TSC) that acts as the oversight body on behalf of the sponsor and funder. At least half of the members, including the chair, are independent. The committee will meet at least once a year. No Data Monitoring and Ethics Committee will be convened for ACCU, as this role will be assumed by the TSC. The role of the Trial Steering Committee is to ensure the trial is on track as set out in the protocol, to ensure the safety of the patients, to help deal with any problems or issues that might arise and to advise the principal investigators and co-investigators. Among the terms of reference of the steering committee, as stipulated in their charter, is to recommend whether to continue or terminate the study or further adapt it based on safety and efficacy considerations.

## Discussion

With the current COVID situation, it was felt best not to set numerical progression criteria which we may fail to attain due to constraints of the COVID-19 lockdowns. However, we will carefully monitor the rate of recruitment and percentage of participants followed up, in order to determine whether progression is feasible and to learn how these could be improved in a full-scale trial. If this trial demonstrates the feasibility of recruitment and delivery, we will seek funding for a fully powered cluster-randomised trial of the intervention. We will use data from the process evaluation (involving potential clients, village health teams and health workers), as well as feedback from district health teams and the Ministry of Health, to optimise the intervention package as well as trial procedures.

The methodology of asking VHTs to collect research data using smartphones is innovative. VHTs have previously been asked to collect routine health data using mobile phones, but this will be the first time that the COSMOS software is used by VHTs to collect research data, which will be uploaded in real time to a secure database. There are a number of challenges: some VHTs have a very basic level of education and patchy mobile phone networks, and some villages do not have electricity. We will test ways of overcoming these barriers. If this system works, it could be of great benefit to a wide variety of researchers collecting data from community-based interventions in Uganda and elsewhere in Africa, making the data collection quicker, cleaner and more secure than traditional paper-based questionnaires.

In order to assess the effectiveness of our intervention in a cluster-randomised trial, there should be no information sharing (“contamination”) between the experiment and control arms. However, with ease in transport and availability of telecommunication services, people in the intervention clusters may share their health information with the control arm population. This feasibility trial will also enable us to ascertain whether “contamination” occurs between clusters.

Understanding the effectiveness of an antenatal couples’ counselling intervention for improving the appropriate place of delivery and uptake of postpartum contraception in Uganda, in a resource-constrained setting like Uganda, is critical for creating public awareness, influencing policy recommendations and developing effective interventions.

### Dissemination plan

We will hold dissemination meetings with stakeholders at the local and national levels to present our results at the end of the project. We will have community meetings to disseminate the findings. We will write up our scientific research in an academic paper which will be submitted for publication in a peer-reviewed scientific journal. We will also submit abstracts to relevant conferences. We will post a summary of our important results on the project website, hosted by the University of Southampton.

### Status of the study

The recruitment and follow-up of participants started in February 2021 and are still ongoing.

## Supplementary Information

**Additional file 1.** ACCU_SPIRIT 2013 Checklist: Recommended items to address in a clinical trial protocol and related documents*.

**Additional file 2.** Participant information sheet and consent form.

**Additional file 3.** ACCU data collection tool.

## Data Availability

At the time of publication, we will make the anonymised datasets freely available on a data repository of the University of Southampton, eprints.soton.ac.uk. In accordance with the University of Southampton’s data policy, the data will be archived from a minimum of 10 years after publication or last access, whichever is longer. DOIs will be issued for the dataset and data subsets as per the University’s DOI policy. As this is a feasibility study, the study team will have exclusive use of data until the end of the follow-on trial as sharing may prejudice the subsequent study and related publications. The data can be accessed by other researchers on reasonable request to the corresponding author at the end of the study.

## Abbreviations

ANC: Antenatal clinic
BP/CR: Birth preparedness and complication readiness
FGDs: Focus group discussions
QALY: Quality-adjusted life year
GPS: Global positioning system
HIV: Human immunodeficiency virus
IUD: Intrauterine device
LARC: Long-acting reversible contraceptive
MoH: Ministry of Health, Uganda
MUREC: Mbarara University of Science and Technology Research Ethics Committee
PNC: Postnatal care
PPFP: Postpartum family planning
RCT: Randomised controlled trial
SBA: Skilled birth attendant
TSC: Trial Steering Committee
UBOS: Uganda Bureau of Statistics
VHTs: Village health teams
WHO: World Health Organization
