# Supplementary material for: Antenatal couples’ counselling in Uganda (ACCU): study protocol for a randomised controlled feasibility trial
Source: Pilot Feasibility Stud. 2022 Apr 29;8:97. doi: 10.1186/s40814-022-01049-5 (PMC9051788; doi:10.1186/s40814-022-01049-5)
Supplement: Supplementary file 2 — Additional file 2. Participant information sheet and consent form. [file 40814_2022_1049_MOESM2_ESM.doc]

**Appendix C.1: Participant information sheet and consent form – for couples in the intervention arm, main study**


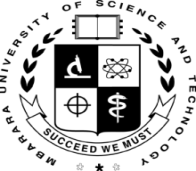
**MBARARA UNIVERSITY OF SCIENCE AND TECHNOLOGY**

**RESEARCH ETHICS COMMITTEE**

**P.O. Box 1410, Mbarara, Uganda**

**Tel. 256-4854-33795 Fax: 256 4854 20782**

**Email:** [**rec@must.ac.ug**](mailto:rec@must.ac.ug) **Web site*:*** [***www.must.ac.ug***](http://www.must.ac.ug/)

**INFORMED CONSENT DOCUMENT**

**Study Title:** ANTENATAL COUPLES’ COUNSELLING IN UGANDA (ACCU)

**Principal Investigator:** Dr. Vincent Mubangizi

**Co-Investigators:**

Mbarara University of Science and Technology: Professor Jerome Kabakyenga, Dr Joseph Ngonzi

University of Southampton, UK: Dr Merlin Willcox, Dr Ingrid Muller, Professor Nuala McGrath, Dr Beth Stuart, Professor James Raftery

**INTRODUCTION**

We would like to invite you to take part in a Research Project about counselling couples during the woman’s pregnancy about the best place to deliver the baby and about family planning after the delivery. We have chosen to invite you to take part because you or your partner is pregnant.

Thank you.

**What you should know about this study:**

- You are being asked to join a research study.
- This consent form explains the research study and your part in the study
- Please read it carefully and take as much time as you need
- You are a volunteer. You can choose not to take part. If you join, you may stop at any time. There will be no penalty if you decide to leave the study.

**Background to the study**

Making good plans for delivery and family planning are both important to keep pregnant mothers and their babies healthy and safe.

**Purpose of the research project:**

We would like to find out if you and your partner will find it helpful to receive counselling together about the best place for your delivery, and about family planning after delivery. We will study the effect this counselling has on your decisions and also on your health.

**Why you are being asked to participate:**

You have been asked to participate because you or your partner is pregnant and we want to study whether counselling will help you to make decisions about your delivery and family planning.

**Procedures:**

A VHT will visit you twice during your pregnancy and will offer you advice about attending the antenatal clinic, choosing a place to deliver, and about family planning after delivery. The VHT will also visit you three times after the delivery (after one week, after 6 months and after 12 months). We will ask you some questions about your pregnancy and family planning at each visit. The whole process will last about 30 minutes on each visit. The VHT will also invite you and your partner to attend antenatal clinic together at the health facility, where a health worker can also offer you counselling.

**Risks / discomforts:**

There are no risks to you from taking part. You are unlikely to feel upset by what we will be asking. We will do our best to respond to your needs during the counselling and questionnaire and will certainly try to avoid upsetting you. You are free not to answer any question or stop the study at any time if you wish.

**Benefits:**

You will benefit from regular follow-up from a VHT who will provide you good advice on your pregnancy and on family planning. You will also benefit from counselling from a health worker. We hope this study will help us improve antenatal and postnatal couples’ counselling locally and elsewhere. You or others in your community might benefit from these improved services in the future.

**Incentives / rewards for participating:**

There are no incentives. We kindly ask you to spare about 30 minutes of your time for us at each of our five visits. We thank you in advance.

**Protecting data confidentiality:**

If you agree to take part, we will ask you to sign a paper consent form. Your name will not be shared with anyone. Only researchers will see the information you provide. Your information will be collected using a smart phone into an online database. All the information we collect will be securely stored, on a password-protected system and computer. The paper forms will be kept in a lockable cupboard.

We will then use what you and others have told us to write a report, but your name will be kept secret and will not appear anywhere. We will share overall results (combining your information with that from other participants) with other professionals and policy makers through meetings. We also hope to publish this report so that other professionals further afield can learn from our project. After removing your name, data collected in this study will be deposited in an online data repository and will be made available to researchers. It will not be possible for you to be identified from this data.

**Protecting subject privacy during data collection:**

Your privacy will be respected. We will ask you the private questions in your own home where no one else can hear your answers.

**Right to refuse / withdraw:**

You are free to decide whether or not you wish to take part. If you choose not to take part you will not be penalised in any way. If you choose to take part, you are free to leave particular questions if you do not want to answer them, or to stop the interview at any time.

**What happens if you leave the study?**

If at any point during the study you decide you do not wish to continue, you are free to ask us to stop. We will respect your wishes and will stop the interview/ contacting you without asking you any more questions or why.

**Who do I ask/call if I have questions or a problem?**

If you have any questions or concerns you can contact us to discuss them using the following details:

Investigators: Dr Vincent Mubangizi, Principal Investigator, ACCU Project, Box 1410, Mbarara, Uganda. Tel: 0772 499 925, Email: [vmubangizi@must.ac.ug](mailto:vmubangizi@must.ac.ug)

Prof Jerome K.Kabakyenga, Co-Investigator ACCU Project, Box 1410, Mbarara, Uganda. Tel: 0772 590 409 Email: [jkabakyenga@must.ac.ug](mailto:jkabakyenga@must.ac.ug)

**Contact for REC office**

Dr. Francis Bajunirwe, Chairman MUST-REC, P.O Box 1410, Mbarara, Tel: 0485433795/ 0772576396

**What does your signature (or thumbprint/mark) on this consent form mean?**

Your signature on this form means

- You have been informed about this study’s purpose, procedures, possible benefits and risks
- You have been given the chance to ask questions before you sign
- You have voluntarily agreed to be in this study
- You agree for anonymised data collected in this study to be deposited in an online data repository and to be made available to researchers. It will not be possible for you to be identified from this data.

----------------------------- ------------------------------ ----------------

Print name of adult participant Signature of adult participant/legally Date

Authorized representative

____________________ __________________ ___________

Print name of person obtaining Signature Date

Consent

**--------------------------------- ------------------------------ -------------------**

Thumbprint/mark signature of witness Date

**Appendix C.2: Participant information sheet and consent form – for couples in the control arm, main study**


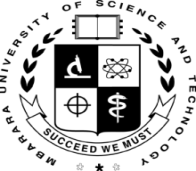
**MBARARA UNIVERSITY OF SCIENCE AND TECHNOLOGY**

**RESEARCH ETHICS COMMITTEE**

**P.O. Box 1410, Mbarara, Uganda**

**Tel. 256-4854-33795 Fax: 256 4854 20782**

**Email:** [**rec@must.ac.ug**](mailto:rec@must.ac.ug) **Web site*:*** [***www.must.ac.ug***](http://www.must.ac.ug/)

**INFORMED CONSENT DOCUMENT**

**Study Title:** ANTENATAL COUPLES’ COUNSELLING IN UGANDA (ACCU)

**Principal Investigator:** Dr. Vincent Mubangizi

**Co-Investigators:**

Mbarara University of Science and Technology: Professor Jerome Kabakyenga, Dr Joseph Ngonzi

University of Southampton, UK: Dr Merlin Willcox, Dr Ingrid Muller, Professor Nuala McGrath, Dr Beth Stuart, Professor James Raftery

**INTRODUCTION**

We would like to invite you to take part in a Research Project about counselling couples during the woman’s pregnancy about the best place to deliver the baby and about family planning after the delivery. We have chosen to invite you to take part because you or your partner is pregnant.

Thank you.

**What you should know about this study:**

- You are being asked to join a research study.
- This consent form explains the research study and your part in the study
- Please read it carefully and take as much time as you need
- You are a volunteer. You can choose not to take part. If you join, you may stop at any time. There will be no penalty if you decide to leave the study.

**Background to the study**

Making good plans for delivery and family planning are both important to keep pregnant mothers and their babies healthy and safe.

**Purpose of the research project:**

We would like to find out if a woman and her partner will find it helpful to receive counselling together about the best place for your delivery, and about family planning after delivery.

We will study the effect that counselling has on their decisions and also on their health.

**Why you are being asked to participate:**

You have been asked to participate because you or your partner is pregnant. We would like to know, in absence of couple counselling, how you make your decisions and how is your health.

**Procedures:**

A VHT will visit you twice during your pregnancy and three times after the delivery (after one week, after 6 months and after 12 months). We will ask you some questions about your pregnancy and family planning at each visit. The whole process will last about 30 minutes on each visit.

**Risks / discomforts:**

There are no risks to you from taking part. You are unlikely to feel upset by what we will be asking. We will do our best to respond to your needs during the administration of the questionnaire and will certainly try to avoid upsetting you. You are free not to answer any question or stop the study at any time if you wish.

**Benefits:**

We hope this study will help us improve antenatal and postnatal couples’ counselling locally and elsewhere. You or others in your community might benefit from these improved services in the future.

**Incentives / rewards for participating:**

There are no incentives. We kindly ask you to spare about 30 minutes of your time for us at each of our five visits. We thank you in advance.

**Protecting data confidentiality:**

If you agree to take part, we will ask you to sign a paper consent form. Your name will not be shared with anyone. Only researchers will see the information you provide. Your information will be collected using a smart phone into an online database. All the information we collect will be securely stored, on a password-protected system and computer. The paper forms will be kept in a lockable cupboard.

We will then use what you and others have told us to write a report, but your name will be kept secret and will not appear anywhere. We will share overall results (combining your information with that from other participants) with other professionals and policy makers through meetings. We also hope to publish this report so that other professionals can learn from our project. After removing your name, data collected in this study will be deposited in an online data repository and will be made available to researchers. It will not be possible for you to be identified from this data.

**Protecting subject privacy during data collection:**

Your privacy will be respected. We will ask you the private questions in your own home where no one else can hear your answers.

**Right to refuse / withdraw:**

You are free to decide whether or not you wish to take part. If you choose not to take part you will not be penalised in any way. If you choose to take part, you are free to leave particular questions if you do not want to answer them, or to stop the interview at any time.

**What happens if you leave the study?**

If at any point during the study you decide you do not wish to continue, you are free to ask us to stop. We will respect your wishes and will stop the interview/ contacting you without asking you any more questions or why.

**Who do I ask/call if I have questions or a problem?**

If you have any questions or concerns you can contact us to discuss them using the following details:

Investigators: Dr Vincent Mubangizi, Principal Investigator, ACCU Project, Box 1410, Mbarara, Uganda. Tel: 0772 499 925, Email: [vmubangizi@must.ac.ug](mailto:vmubangizi@must.ac.ug)

Prof Jerome Kabakyenga, Co-Investigator ACCU Project, Box 1410, Mbarara, Uganda. Tel: 0772 590 409 Email: [jkabakyenga@must.ac.ug](mailto:jkabakyenga@must.ac.ug)

- **Contact for REC office**

Dr. Francis Bajunirwe, Chairman MUST-REC, P.O Box 1410, Mbarara, Tel: 0485433795/ 0772 576396

**What does your signature (or thumbprint/mark) on this consent form mean?**

Your signature on this form means

- You have been informed about this study’s purpose, procedures, possible benefits and risks
- You have been given the chance to ask questions before you sign
- You have voluntarily agreed to be in this study
- You agree for anonymised data collected in this study to be deposited in an online data repository and to be made available to researchers. It will not be possible for you to be identified from this data.

----------------------------- ------------------------------ ----------------

Print name of adult participant Signature of adult participant/legally Date

Authorized representative

____________________ __________________ ___________

Print name of person obtaining Signature Date

Consent

**--------------------------------- ------------------------------ -------------------**

Thumbprint/mark signature of witness Date

# Appendix C.3: Participant Information Sheet and Consent Form – for Health Service Providers – individual qualitative interviews


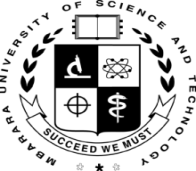
**MBARARA UNIVERSITY OF SCIENCE AND TECHNOLOGY**

**RESEARCH ETHICS COMMITTEE**

**P.O. Box 1410, Mbarara, Uganda**

**Tel. 256-4854-33795 Fax: 256 4854 20782**

**Email:** [**rec@must.ac.ug**](mailto:rec@must.ac.ug) **Web site*:*** [***www.must.ac.ug***](http://www.must.ac.ug/)

**INFORMED CONSENT DOCUMENT**

**Study Title:** ANTENATAL COUPLES’ COUNSELLING IN UGANDA (ACCU)

**Principal Investigator(s):** Dr. Vincent Mubangizi

**Co-Investigators:**

Mbarara University of Science and Technology: Professor Jerome Kabakyenga, Dr Joseph Ngonzi

University of Southampton, UK: Dr Merlin Willcox, Dr Ingrid Muller, Professor Nuala McGrath, Dr Beth Stuart, Professor James Raftery

**INTRODUCTION**

Hello, I am ………………………………, a member of the research team from the Mbarara University of Science and Technology. We would like to invite your participation in a Research Project that seeks to find out whether it is acceptable and feasible to provide antenatal and post natal couples’ counselling intervention for improving appropriate place of delivery and uptake of postpartum contraception in Uganda, This study is very important for improving antenatal and post-natal couples’ counselling for post-partum family planning and birth planning locally and elsewhere.

We have purposefully chosen to invite you to participate because you are a health worker involved in providing couples’ counselling.

Thank you.

**What you should know about this study:**

- You are being asked to join a research study.
- This consent form explains the research study and your part in the study
- Please read it carefully and take as much time as you need
- You are a volunteer. You can choose not to take part and if you join, you may quit at any time. There will be no penalty if you decide to quit the study

**Background to the study**

In Uganda, maternal, perinatal and neonatal deaths remain high. The common avoidable factors leading to death include lack of birth planning (and delivery in an inappropriate place) and unmet need for contraception. Although some health workers have been trained to provide postpartum family planning, uptake remains low because most women first want the approval of their husbands, who are usually absent at the time of delivery. One way to overcome these barriers is to improve information and counselling for couples on post-partum family planning and birth planning during antenatal and post natal clinics. We have been running a project to improve provision of antenatal couples’ counselling, in which your health facility has taken part.

**Purpose of the research project:**

We would like to find out what you think of the antenatal and postnatal couples’ counselling intervention for improving appropriate place of delivery and uptake of postpartum contraception, so that we improve the delivery of intervention. We will record the discussion on a digital voice recorder. The discussion will last about 45 minutes- to one hour.

**Why you are being asked to participate:**

You have been asked to participate because you are a health worker who has been involved in the project to improve delivery of antenatal and post natal couples’ counselling on post-partum family planning and birth planning. We would like to hear about your experiences of the training, taking part in the research and your views on the implementation of antenatal and post natal couples’ counselling on post-partum family planning and birth planning.

**Procedures:**

We will be having a discussion which will be recorded with a digital voice recorder. The discussion will last 45 minutes to one hour depending on how much you want to share with us.

**Risks / discomforts:**

There are no physical risks. You unlikely to feel upset by what we will be asking. We will do our best to respond to your needs during the interview and will certainly try to avoid upsetting you. You are free not to answer any question or stop the interview at any time if you wish.

**Benefits:**

We hope this study will help us improve antenatal and postnatal couples’ counselling for post-partum family planning services and birth planning locally and elsewhere. You or others in your community might benefit from these improved services in the future.

**Incentives / rewards for participating:**

We will provide refreshments during the interview and will refund your travel costs/compensate or your time at ten thousand shillings only (10,000 UGX).

**Protecting data confidentiality:**

You shall be asked to fill in paper consent forms separately and no identification is required. The identifiable data will be coded and the ‘links’ kept separate. We will record our discussion using an audio recorder, then write down what was said. Only researchers will have access to the raw data. All collected data and personal information about the participants will be securely stored, on a password-protected system and computer. The paper consent forms will be kept in a lockable cupboard. Your name will not be mentioned, so no one else will know what you have told us. The voice recordings will be destroyed. We will then use what you and others have told us to write a report. We will share our results with other professionals and policy makers through meetings. We also hope to publish this report so that other professionals further afield can learn from your experiences. After removing your name, data collected in this study will be deposited in an online data repository and will be made available to researchers. It will not be possible for you to be identified from this data.

**Protecting subject privacy during data collection:**

Subject privacy will be respected. Interviews will be done in a place where there will be no intruders.

**Right to refuse / withdraw:**

You are free to decide whether or not you wish to take part. If you choose not to take part you will not be penalised in any way. If you choose to take part, you are free to leave particular questions if you do not want to answer them, or to stop the interview at any time.

**What happens if you leave the study?**

If at any point during the study you decide you do not wish to continue, you are free to ask us to stop. We will respect your wishes and will stop the interview without asking you any more questions.

**Who do I ask/call if I have questions or a problem?**

If you have any questions or concerns you can contact us to discuss them using the following details:

Investigators: Dr Vincent Mubangizi, Principal Investigator, ACCU Project, Box 1410, Mbarara, Uganda. Tel: 0772 499 925, Email: [vmubangizi@must.ac.ug](mailto:vmubangizi@must.ac.ug)

Prof Jerome K.Kabakyenga, Co-Investigator ACCU Project, Box 1410, Mbarara, Uganda. Tel: 0772 590 409 Email: [jkabakyenga@must.ac.ug](mailto:jkabakyenga@must.ac.ug)

- **Contact for REC office**

Dr. Francis Bajunirwe, Chairman MUST-REC, P.O Box 1410, Mbarara, Tel: 0485433795 /0772 576 396

**What does your signature (or thumbprint/mark) on this consent form mean?**

Your signature on this form means

- You have been informed about this study’s purpose, procedures, possible benefits and risks
- You have been given the chance to ask questions before you sign
- You have voluntarily agreed to be in this study
- You agree for anonymised data collected in this study to be deposited in an online data repository and to be made available to researchers. It will not be possible for you to be identified from this data.

------------------------------- --------------------------- --------------

Print name of adult participant Signature of adult participant/legally Date

Authorized representative

_____________________ _________________ ___________

Print name of person obtaining Signature Date

Consent

**Appendix C.4: Participant Information Sheet and Consent Form – for clinic managers for in depth interview**


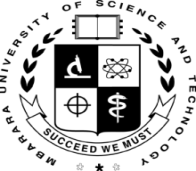
**MBARARA UNIVERSITY OF SCIENCE AND TECHNOLOGY**

**RESEARCH ETHICS COMMITTEE**

**P.O. Box 1410, Mbarara, Uganda**

**Tel. 256-4854-33795 Fax: 256 4854 20782**

**Email:** [**rec@must.ac.ug**](mailto:rec@must.ac.ug) **Web site*:*** [***www.must.ac.ug***](http://www.must.ac.ug/)

**INFORMED CONSENT DOCUMENT**

**Study Title:** ANTENATAL COUPLES’ COUNSELLING IN UGANDA (ACCU)

**Principal Investigator(s):** Dr. Vincent Mubangizi

**Co-Investigators:**

Mbarara University of Science and Technology: Professor Jerome Kabakyenga, Dr Joseph Ngonzi

University of Southampton, UK: Dr Merlin Willcox, Dr Ingrid Muller, Professor Nuala McGrath, Dr Beth Stuart, Professor James Raftery

**INTRODUCTION**

Hello, I am ……………………………… a member of the research team from the Mbarara University of Science and Technology. We would like to invite your participation in a Research Project that seeks to find out whether it is acceptable and feasible to provide antenatal and post natal couples’ counselling intervention for improving appropriate place of delivery and uptake of postpartum contraception in Uganda. This study is very important for improving antenatal and post-natal couples’ counselling for post-partum family planning and birth planning locally and elsewhere.

We have purposefully chosen to invite you to participate because you are a clinic manager involved in our project about providing couples’ counselling. Thank you.

**What you should know about this study:**

- You are being asked to join a research study.
- This consent form explains the research study and your part in the study
- Please read it carefully and take as much time as you need
- You are a volunteer. You can choose not to take part and if you join, you may quit at any time. There will be no penalty if you decide to quit the study.

**Background to the study**

In Uganda, maternal, perinatal and neonatal deaths remain high. The common avoidable factors leading to death include lack of birth planning (and delivery in an inappropriate place) and unmet need for contraception. Although some health workers have been trained to provide postpartum family planning, uptake remains low because most women first want the approval of their husbands, who are usually absent at the time of delivery. One way to overcome these barriers is to improve information and counselling for couples on post-partum family planning and birth planning during antenatal and post natal clinics. We have been running a project to improve provision of antenatal couples’ counselling, in which your health facility has taken part.

**Purpose of the research project:**

We would like to find out what you think our intervention to improve antenatal and post natal couples’ counselling for increasing use of an appropriate place of delivery and uptake of postpartum contraception in Uganda. Your feedback will help us to improve the intervention. We will record the discussion on a digital voice recorder. The discussion will last about 45minutes to one hour.

**Why you are being asked to participate:**

You have been asked to participate because you are a clinic manager who is involved in leadership and management of the health facility taking part in this project. You provide oversight to ensure delivery of antenatal and post natal couples’ counselling on post-partum family planning and birth planning. We would like to hear your views on implementation of antenatal and post natal couples’ counselling on post-partum family planning and birth planning.

**Procedures:**

We will be having a discussion which will be recorded with a tape recorder. The discussion will last 45 minutes to one hour depending on how much you want to share with us.

**Risks / discomforts:**

You are unlikely to feel upset by what we will be asking. We will do our best to respond to your needs during the interview and will certainly try to avoid upsetting you. You are free not to answer any question or stop the interview at any time if you wish.

**Benefits:**

We hope this study will help us improve antenatal and postnatal couples’ counselling for post-partum family planning services and birth planning locally and elsewhere. You or others in your community might benefit from these improved services in the future.

**Incentives / rewards for participating:**

We will provide refreshments during the interview and will refund your travel costs/ compensate for your time at ten thousand shillings only (10,000 UGX).

**Protecting data confidentiality:**

You shall be asked to fill in a paper consent form separately and no identification is required. The identifiable data will be coded and the ‘links’ kept separate. We will record our discussion using an audio recorder, then write down what was said. Only researchers will have access to the raw data. All collected data and personal information about the participants will be securely stored, on a password-protected system and computer. The hard copies will be kept in a lockable cupboard. Your name will not be mentioned, so no one else will know what you have told us. The voice recordings will be destroyed. We will then use what you and others have told us to write a report. We will share our results with other professionals and policy makers through meetings. We also hope to publish this report so that other professionals further afield can learn from our project. After removing your name, data collected in this study will be deposited in an online data repository and will be made available to researchers. It will not be possible for you to be identified from this data.

**Protecting subject privacy during data collection:**

Subject privacy will be respected. Interviews will be done in a place where there will be no intruders.

**Right to refuse / withdraw:**

You are free to decide whether or not you wish to take part. If you choose not to take part you will not be penalised in any way. If you choose to take part, you are free to leave particular questions if you do not want to answer them, or to stop the interview at any time.

**What happens if you leave the study?**

If at any point during the study you decide you do not wish to continue, you are free to ask us to stop. We will respect your wishes and will stop the interview without asking you any more questions.

**Who do I ask/call if I have questions or a problem?**

If you have any questions or concerns you can contact us to discuss them using the following details:

Investigators: Dr Vincent Mubangizi, Principal Investigator, ACCU Project, Box 1410, Mbarara, Uganda. Tel: 0772 499 925, Email: [vmubangizi@must.ac.ug](mailto:vmubangizi@must.ac.ug)

Prof Jerome K.Kabakyenga, Co-Investigator ACCU Project, Box 1410, Mbarara, Uganda. Tel: 0772 590 409 Email: [jkabakyenga@must.ac.ug](mailto:jkabakyenga@must.ac.ug)

- **Contact for REC office**

Dr. Francis Bajunirwe, Chairman MUST-REC, P.O Box 1410, Mbarara, Tel: 0485433795 /0772 576 396

**What does your signature (or thumbprint/mark) on this consent form mean?**

Your signature on this form means

- You have been informed about this study’s purpose, procedures, possible benefits and risks
- You have been given the chance to ask questions before you sign
- You have voluntarily agreed to be in this study
- You agree for anonymised data collected in this study to be deposited in an online data repository and to be made available to researchers. It will not be possible for you to be identified from this data.

--------------------------------- ----------------------------- ---------------

Print name of adult participant Signature of adult participant/legally Date

Authorized representative

____________________ _________________ _________

Print name of person obtaining Signature Date

Consent

**Appendix C.5: Participant Information Sheet and Consent Form – Village health team members (VHTs) for in-depth interview**


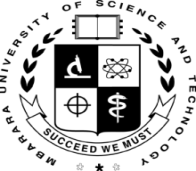
**MBARARA UNIVERSITY OF SCIENCE AND TECHNOLOGY**

**RESEARCH ETHICS COMMITTEE**

**P.O. Box 1410, Mbarara, Uganda**

**Tel. 256-4854-33795 Fax: 256 4854 20782**

**Email:** [**rec@must.ac.ug**](mailto:rec@must.ac.ug) **Web site*:*** [***www.must.ac.ug***](http://www.must.ac.ug/)

**INFORMED CONSENT DOCUMENT**

**Study Title:** ANTENATAL COUPLES’ COUNSELLING IN UGANDA (ACCU)

**Principal Investigator(s):** Dr. Vincent Mubangizi

**Co-Investigators:**

Mbarara University of Science and Technology: Professor Jerome Kabakyenga, Dr Joseph Ngonzi

University of Southampton, UK: Dr Merlin Willcox, Dr Ingrid Muller, Professor Nuala McGrath, Dr Beth Stuart, Professor James Raftery

**INTRODUCTION**

Hello, I am ……………………………… a member of the research team from the Mbarara University of Science and Technology. We would like to invite your participation in a Research Project that seeks to find out whether it is acceptable and feasible to provide antenatal and post natal couples’ counselling intervention for improving appropriate place of delivery and uptake of postpartum contraception in Uganda, This study is very important for improving antenatal and post-natal couples’ counselling for post-partum family planning and birth planning locally and elsewhere.

We have purposefully chosen to invite you to participate because you are a village health team (VHT) member involved in the couples’ counselling project. Thank you.

**What you should know about this study:**

- You are being asked to join a research study.
- This consent form explains the research study and your part in the study
- Please read it carefully and take as much time as you need
- You are a volunteer. You can choose not to take part and if you join, you may quit at any time. There will be no penalty if you decide to quit the study

**Background to the study**

Making good plans for delivery and family planning are both important to keep pregnant mothers and their babies healthy and safe. Providing counselling to couples may help them to make better plans about where the baby is delivered, and about family planning after the delivery.

**Purpose of the research project:**

We would like to find out what you think of the study on antenatal and postnatal couples’ counselling to increase the appropriate place of delivery and uptake of postpartum contraception in Uganda. Your feedback will help us to improve the delivery of the intervention.

We would like to invite you to be interviewed on your own about your experiences of the project.

**Why you are being asked to participate:**

You have been asked to participate because you are a VHT involved in delivery of antenatal and post natal couples’ counselling on post-partum family planning and birth planning. We would like to hear your experiences of taking part in this research project, and your views on implementation of antenatal and post natal couples’ counselling.

**Procedures:**

We will ask you the questions in a private place where no one else can hear your answers. The interview will be recorded with a digital voice recorder and will last 45 minutes to one hour depending on how much you want to share with us.

**Risks / discomforts:**

You are unlikely to feel upset by what we will be asking. We will do our best to respond to your needs during the interview and will certainly try to avoid upsetting you. You are free not to answer any question or stop the interview at any time if you wish.

**Benefits:**

We hope this study will help us improve antenatal and postnatal couples’ counselling for post-partum family planning services and birth planning locally and elsewhere. You or others in your community might benefit from these improved services in the future.

**Incentives / rewards for participating:**

We will provide refreshments during the interview and will refund your travel costs/compensate for your time at ten thousand shillings only (10,000 UGX).

**Protecting data confidentiality:**

You shall be asked to fill in a paper consent form separately and no identification is required. The identifiable data will be coded and the ‘links’ kept separate. We will record our discussion using an audio recorder, then write down what was said.

Only researchers will have access to the raw data. All collected data and personal information about the participants will be securely stored, on a password-protected system and computer. The paper copies will be kept in a lockable cupboard. Your name will not be mentioned, so no one else will know what you have told us. The voice recordings will be destroyed. We will then use what you and others have told us to write a report. We will share our results with other professionals and policy makers through meetings. We also hope to publish this report so that other professionals further afield can learn about the situation here. After removing your name, data collected in this study will be deposited in an online data repository and will be made available to researchers. It will not be possible for you to be identified from this data.

**Protecting subject privacy during data collection:**

Your privacy will be respected. Interviews will be done in a place where there is no one else.

**Right to refuse / withdraw:**

You are free to decide whether or not you wish to take part. If you choose not to take part you will not be penalised in any way. If you choose to take part, you are free to leave particular questions if you do not want to answer them, or to stop the interview at any time.

**What happens if you leave the study?**

If at any point during the study you decide you do not wish to continue, you are free to ask us to stop. We will respect your wishes and will stop the interview/ contacting you without asking you any more questions or why.

**Who do I ask/call if I have questions or a problem?**

If you have any questions or concerns you can contact us to discuss them using the following details:

Investigators: Dr Vincent Mubangizi, Principal Investigator, ACCU Project, Box 1410, Mbarara, Uganda. Tel: 0772 499 925, Email: [vmubangizi@must.ac.ug](mailto:vmubangizi@must.ac.ug)

Prof Jerome Kabakyenga, Co-Investigator ACCU Project, Box 1410, Mbarara, Uganda. Tel: 0772 590 409 Email: [jkabakyenga@must.ac.ug](mailto:jkabakyenga@must.ac.ug)

**Contact for REC office**

Dr. Francis Bajunirwe, Chairman MUST-REC, P.O Box 1410, Mbarara, Tel: 0485433795 /0772 576 396

**What does your signature (or thumbprint/mark) on this consent form mean?**

Your signature on this form means

- You have been informed about this study’s purpose, procedures, possible benefits and risks
- You have been given the chance to ask questions before you sign
- You have voluntarily agreed to be in this study
- You agree for anonymised data collected in this study to be deposited in an online data repository and to be made available to researchers. It will not be possible for you to be identified from this data.

-------------------------- -------------------------- ----------

Print name of adult participant Signature of adult participant/legally Date

Authorized representative

_________________ __________________ ________

Print name of person obtaining consent Signature Date

# Appendix C.6: Participant Information Sheet and Consent Form – couples, men, adult women and adolescent women for in-depth interview


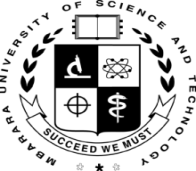
**MBARARA UNIVERSITY OF SCIENCE AND TECHNOLOGY**

**RESEARCH ETHICS COMMITTEE**

**P.O. Box 1410, Mbarara, Uganda**

**Tel. 256-4854-33795 Fax: 256 4854 20782**

**Email:** [**rec@must.ac.ug**](mailto:rec@must.ac.ug) **Web site*:*** [***www.must.ac.ug***](http://www.must.ac.ug/)

**INFORMED CONSENT DOCUMENT**

**Study Title:** ANTENATAL COUPLES’ COUNSELLING IN UGANDA (ACCU)

**Principal Investigator(s):** Dr. Vincent Mubangizi

**Co-Investigators:**

Mbarara University of Science and Technology: Professor Jerome Kabakyenga, Dr Joseph Ngonzi

University of Southampton, UK: Dr Merlin Willcox, Dr Ingrid Muller, Professor Nuala McGrath, Dr Beth Stuart, Professor James Raftery

**INTRODUCTION**

Hello, I am ………………………………a member of a research team from the Mbarara University of Science and Technology. You have already agreed to take part in the Research Project about couples’ counselling in the antenatal clinic. Now we would also like to invite you to take part in an interview about your experiences of this study. This is very important for improving the couples’ counselling locally and elsewhere and for improving the project.

We have purposefully chosen to invite you to participate because you and/or your partner took part in the project. Thank you.

**What you should know about this study:**

- You are being asked to join a research study.
- This consent form explains the research study and your part in the study
- Please read it carefully and take as much time as you need
- You are a volunteer. You can choose not to take part and if you join, you may quit at any time. There will be no penalty if you decide to quit the study

**Background to the study**

Making good plans for delivery and family planning are both important to keep pregnant mothers and their babies healthy and safe. You have been taking part in our project on counselling for couples on post-partum family planning and birth planning. We would like to learn from your experiences of taking part in the project, so that we can improve it in the future.

**Purpose of the research project:**

We would like to find out about your experiences of taking part in the study on antenatal and post natal couples’ counselling.

We would like to invite you to be interviewed on your own. We will ask you the questions in a private place where no one else can hear your answers.

**Why you are being asked to participate:**

We have purposefully chosen to invite you to participate because you and/or your partner have taken part in the project on antenatal and post natal couples’ counselling. We would like to hear your experiences of the project and your views on how it could be improved.

**Procedures:**

A researcher will ask you some questions about your experiences of the project. This interview will be recorded with a digital voice recorder. The interview will last 45 minutes to one hour depending on how much you want to share with us.

**Risks / discomforts:**

You are unlikely to feel upset by what we will be asking. We will do our best to respond to your needs during the interview and will certainly try to avoid upsetting you. You are free not to

answer any question or to stop the interview at any time if you wish.

**Benefits:**

We hope this study will help us to improve antenatal and postnatal couples’ counselling locally and elsewhere. You or others in your community might benefit from these improved services in the future.

**Incentives / rewards for participating:**

We will provide refreshments during the interview and will refund your travel costs/compensate for your time at ten thousand shillings only (10,000 UGX).

**Protecting data confidentiality:**

You shall be asked to fill in paper consent forms separately and no identification is required. The identifiable data will be coded and the ‘links’ kept separate. We will record our discussion using an audio recorder, then write down what was said. We will ask all the participants to treat the discussion as confidential. We also ask you to keep all information confidential regarding other participants.

Only researchers will have access to the raw data. All collected data and personal information will be securely stored, on a password-protected system and computer. The paper copies will be kept in a lockable cupboard. Your name will not be mentioned, so no one else will know what you have told us. The voice recordings will be destroyed.

We will then use what you and others have told us to write a report. We will share our results with other professionals and policy makers through meetings. We also hope to publish this report so that other professionals further afield can learn about the situation here. After removing your name, data collected in this study will be deposited in an online data repository and will be made available to researchers. It will not be possible for you to be identified from this data.

**Protecting subject privacy during data collection:**

Subject privacy will be respected. Interviews will be done in a place where there will be no intruders.

**Right to refuse / withdraw:**

You are free to decide whether or not you wish to take part. If you choose not to take part, you will not be penalised in any way. If you choose to take part, you are free to leave particular questions if you do not want to answer them, or to stop the interview at any time.

**What happens if you leave the study?**

If at any point during the study you decide you do not wish to continue, you are free to ask us to stop. We will respect your wishes and will stop the interview without asking you any more questions.

**Who do I ask/call if I have questions or a problem?**

If you have any questions or concerns you can contact us to discuss them using the following details:

Investigators: Dr Vincent Mubangizi, Principal Investigator, ACCU Project, Box 1410, Mbarara, Uganda. Tel: 0772 499 925, Email: [vmubangizi@must.ac.ug](mailto:vmubangizi@must.ac.ug)

Prof Jerome Kabakyenga, Co-Investigator ACCU Project, Box 1410, Mbarara, Uganda. Tel: 0772 590 409 Email: [jkabakyenga@must.ac.ug](mailto:jkabakyenga@must.ac.ug)

**Contact for REC office**

Dr. Francis Bajunirwe, Chairman MUST-REC, P.O Box 1410, Mbarara, Tel: 0485433795 / 0772 576 396

**What does your signature (or thumbprint/mark) on this consent form mean?**

Your signature on this form means

- You have been informed about this study’s purpose, procedures, possible benefits and risks
- You have been given the chance to ask questions before you sign
- You have voluntarily agreed to be in this study
- You agree for anonymised data collected in this study to be deposited in an online data repository and to be made available to researchers. It will not be possible for you to be identified from this data.

--------------------------- ------------------------- ------------

Print name of adult participant Signature of adult participant/legally Date

Authorized representative

__________________ ________________ ________

Print name of person obtaining Signature Date

Consent

**----------------------------- -------------------------- ------------**

Thumb print/mark signature of witness Date

# Appendix C.7: Participant Information Sheet and Consent Form – couples, men, adult women and adolescent women for focus group discussion


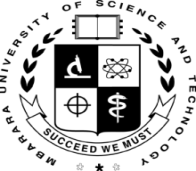
**MBARARA UNIVERSITY OF SCIENCE AND TECHNOLOGY**

**RESEARCH ETHICS COMMITTEE**

**P.O. Box 1410, Mbarara, Uganda**

**Tel. 256-4854-33795 Fax: 256 4854 20782**

**Email:** [**rec@must.ac.ug**](mailto:rec@must.ac.ug) **Web site*:*** [***www.must.ac.ug***](http://www.must.ac.ug/)

**INFORMED CONSENT DOCUMENT**

**Study Title:** ANTENATAL COUPLES’ COUNSELLING IN UGANDA (ACCU)

**Principal Investigator(s):** Dr. Vincent Mubangizi

**Co-Investigators:**

Mbarara University of Science and Technology: Professor Jerome Kabakyenga, Dr Joseph Ngonzi

University of Southampton, UK: Dr Merlin Willcox, Dr Ingrid Muller, Professor Nuala McGrath, Dr Beth Stuart, Professor James Raftery

**INTRODUCTION**

Hello, I am ………………………………a member of a research team from the Mbarara University of Science and Technology. You have already agreed to take part in the Research Project about couples’ counselling in the antenatal clinic. Now we would also like to invite you to take part in a focus group discussion about your experiences of this study. This is very important for improving the couples’ counselling locally and elsewhere and for improving the project. Thank you.

**What you should know about this study:**

- You are being asked to join a research study.
- This consent form explains the research study and your part in the study
- Please read it carefully and take as much time as you need
- You are a volunteer. You can choose not to take part and if you join, you may quit at any time. There will be no penalty if you decide to quit the study.

**Background to the study**

Making good plans for delivery and family planning are both important to keep pregnant mothers and their babies healthy and safe. You have been taking part in our project on counselling for couples on post-partum family planning and birth planning. We would like to learn from your experiences of taking part in the project, so that we can improve it in the future.

**Purpose of the research project:**

We would like to find out about your experiences of taking part in the study on antenatal and postnatal couples’ counselling.

**Why you are being asked to participate:**

We have purposefully chosen to invite you to participate because you and/or your partner have taken part in the project on antenatal and postnatal couples’ counselling. We would like to hear your experiences of the project and your views on how it could be improved.

**Procedures:**

We would like to invite you to join a group discussion with about 10 other people. This will be recorded with a digital voice recorder. The discussion will last about one hour depending on how much you want to share with us.

**Risks / discomforts:**

You are unlikely to feel upset by what we will be asking. We will do our best to respond to your needs during the discussion and will certainly try to avoid upsetting you. You are free not to

answer any question or to leave the group discussion at any time if you wish. The other members of the group discussion will hear what you share. Please do not share what you hear with anyone else outside the group.

**Benefits:**

We hope this study will help us to improve antenatal and postnatal couples’ counselling locally and elsewhere. You or others in your community might benefit from these improved services in the future.

**Incentives / rewards for participating:**

We will provide refreshments during the interview and will refund your travel costs/compensate for your time at ten thousand shillings only (10,000 UGX).

**Protecting data confidentiality:**

We will ask you to sign this consent form. No identification is required. The identifiable data will be coded and the ‘links’ kept separate. We will record our discussion using an audio recorder, then write down what was said. We will ask all the participants to treat the discussion as confidential. We also ask you to keep all information confidential regarding other participants.

Only researchers will have access to the raw data. All collected data and personal information about the participants will be securely stored, on a password-protected system and computer. The paper copies will be kept in a lockable cupboard. Your name will not be mentioned, so no one else will know what you have told us. The voice recordings will be destroyed.

We will then use what you and others have told us to write a report. We will share our results with other professionals and policy makers through meetings. We also hope to publish this report so that other professionals further afield can learn about the situation here. After removing your name, data collected in this study will be deposited in an online data repository and will be made available to researchers. It will not be possible for you to be identified from this data.

**Protecting subject privacy during data collection:**

We will respect your privacy. The discussions will be held in a place where there will be no intruders.

**Right to refuse / withdraw:**

You are free to decide whether or not you wish to take part. If you choose not to take part you will not be penalised in any way. If you choose to take part, you are free to leave particular questions if you do not want to answer them, or to step out from the group discussion at any time.

**What happens if you leave the study?**

If at any point during the discussion you decide you do not wish to continue, you are free to step out. We will respect your wishes and will continue the discussion without you.

**Who do I ask/call if I have questions or a problem?**

If you have any questions or concerns you can contact us to discuss them using the following details:

Investigators: Dr Vincent Mubangizi, Principal Investigator, ACCU Project, Box 1410, Mbarara, Uganda. Tel: 0772 499 925, Email: [vmubangizi@must.ac.ug](mailto:vmubangizi@must.ac.ug)

Prof Jerome Kabakyenga, Co-Investigator ACCU Project, Box 1410, Mbarara, Uganda. Tel: 0772 590 409 Email: [jkabakyenga@must.ac.ug](mailto:jkabakyenga@must.ac.ug)

**Contact for REC office**

Dr. Francis Bajunirwe, Chairman MUST-REC, P.O Box 1410, Mbarara, Tel: 0485433795 / 0772 576 396

**What does your signature (or thumbprint/mark) on this consent form mean?**

Your signature on this form means

- You have been informed about this study’s purpose, procedures, possible benefits and risks
- You have been given the chance to ask questions before you sign
- You have voluntarily agreed to be in this study
- You agree for anonymised data collected in this study to be deposited in an online data repository and to be made available to researchers. It will not be possible for you to be identified from this data.

--------------------------- ------------------------- ------------

Print name of adult participant Signature of adult participant/legally Date

Authorized representative

__________________ ________________ ________

Print name of person obtaining Signature Date

Consent

**----------------------------- -------------------------- ------------**

Thumb print/mark signature of witness Date

**Appendix C.8: Participant Information Sheet and Consent Form – Village health team members (VHTs) for focus group discussion**


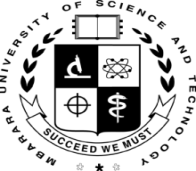
**MBARARA UNIVERSITY OF SCIENCE AND TECHNOLOGY**

**RESEARCH ETHICS COMMITTEE**

**P.O. Box 1410, Mbarara, Uganda**

**Tel. 256-4854-33795 Fax: 256 4854 20782**

**Email:** [**rec@must.ac.ug**](mailto:rec@must.ac.ug) **Web site*:*** [***www.must.ac.ug***](http://www.must.ac.ug/)

**INFORMED CONSENT DOCUMENT**

**Study Title:** ANTENATAL COUPLES’ COUNSELLING IN UGANDA (ACCU)

**Principal Investigator(s):** Dr. Vincent Mubangizi

**Co-Investigators:**

Mbarara University of Science and Technology: Professor Jerome Kabakyenga, Dr Joseph Ngonzi

University of Southampton, UK: Dr Merlin Willcox, Dr Ingrid Muller, Professor Nuala McGrath, Dr Beth Stuart, Professor James Raftery

**INTRODUCTION**

Hello, I am ……………………………… a member of the research team from the Mbarara University of Science and Technology. We would like to invite your participation in a focus group discussion about antenatal and post natal couples’ counselling intervention for improving appropriate place of delivery and uptake of postpartum contraception in Uganda. This study is very important for improving antenatal and post-natal couples’ counselling for post-partum family planning and birth planning locally and elsewhere.

We have purposefully chosen to invite you to participate because you are a village health team (VHT) member involved in the couples’ counselling project. Thank you.

**What you should know about this study:**

- You are being asked to join a research study.
- This consent form explains the research study and your part in the study
- Please read it carefully and take as much time as you need
- You are a volunteer. You can choose not to take part and if you join, you may quit at any time. There will be no penalty if you decide to quit the study

**Background to the study**

Making good plans for delivery and family planning are both important to keep pregnant mothers and their babies healthy and safe. Providing counselling to couples may help them to make better plans about where the baby is delivered, and about family planning after the delivery.

**Purpose of the research project:**

We would like to find out what you think of the study on antenatal and postnatal couples’ counselling to increase the appropriate place of delivery and uptake of postpartum contraception in Uganda. Your feedback will help us to improve the delivery of the intervention.

**Why you are being asked to participate:**

You have been asked to participate because you are a VHT and you have been involved in antenatal and postnatal couples’ counselling on post-partum family planning and birth planning. We would like to hear your experiences of taking part in this research project, and your views on implementation of antenatal and postnatal couples’ counselling.

**Procedures:**

We would like to invite you to join a group discussion with about 10 other people. This will be recorded with a digital voice recorder. The discussion will last about one hour depending on how much you want to share with us.

**Risks / discomforts:**

You are unlikely to feel upset by what we will be asking. We will do our best to respond to your needs during the discussion and will certainly try to avoid upsetting you. You are free not to

answer any question or to leave the group discussion at any time if you wish. The other members of the group discussion will hear what you share. Please do not share what you hear with anyone else outside the group.

**Benefits:**

We hope this study will help us improve antenatal and postnatal couples’ counselling for post-partum family planning services and birth planning locally and elsewhere. You or others in your community might benefit from these improved services in the future.

**Incentives / rewards for participating:**

We will provide refreshments during the discussion and will refund your travel costs/compensate for your time at ten thousand shillings only (10,000 UGX).

**Protecting data confidentiality:**

We will invite you to fill in a paper consent form separately. No identification is required. The identifiable data will be coded and the ‘links’ kept separate. We will record our discussion using an audio recorder, then write down what was said. We will ask all the participants to treat the discussion as confidential. We also ask you to keep all information confidential regarding other participants.

Only researchers will have access to the raw data. All collected data and personal information about the participants will be securely stored, on a password-protected system and computer. The paper copies will be kept in a lockable cupboard. Your name will not be mentioned, so no one else will know what you have told us. The voice recordings will be destroyed. We will then use what you and others have told us to write a report. We will share our results with other professionals and policy makers through meetings. We also hope to publish this report so that other professionals further afield can learn about the situation here. After removing your name, data collected in this study will be deposited in an online data repository and will be made available to researchers. It will not be possible for you to be identified from this data.

**Protecting subject privacy during data collection:**

We will respect your privacy. The discussions will be held in a place where there will be no intruders.

**Right to refuse / withdraw:**

You are free to decide whether or not you wish to take part. If you choose not to take part you will not be penalised in any way. If you choose to take part, you are free to leave particular questions if you do not want to answer them, or to step out from the group discussion at any time.

**What happens if you leave the study?**

If at any point during the discussion you decide you do not wish to continue, you are free to step out. We will respect your wishes and will continue the discussion without you.

**Who do I ask/call if I have questions or a problem?**

If you have any questions or concerns you can contact us to discuss them using the following details:

Investigators: Dr Vincent Mubangizi, Principal Investigator, ACCU Project, Box 1410, Mbarara, Uganda. Tel: 0772 499 925, Email: [vmubangizi@must.ac.ug](mailto:vmubangizi@must.ac.ug)

Prof Jerome Kabakyenga, Co-Investigator ACCU Project, Box 1410, Mbarara, Uganda. Tel: 0772 590 409 Email: [jkabakyenga@must.ac.ug](mailto:jkabakyenga@must.ac.ug)

- **Contact for REC office**

Dr. Francis Bajunirwe, Chairman MUST-REC, P.O Box 1410, Mbarara, Tel: 0485433795 /0772 576 396

**What does your signature (or thumbprint/mark) on this consent form mean?**

Your signature on this form means

- You have been informed about this study’s purpose, procedures, possible benefits and risks
- You have been given the chance to ask questions before you sign
- You have voluntarily agreed to be in this study
- You agree for anonymised data collected in this study to be deposited in an online data repository and to be made available to researchers. It will not be possible for you to be identified from this data.

-------------------------- -------------------------- ----------

Print name of adult participant Signature of adult participant/legally Date

Authorized representative

_________________ __________________ ________

Print name of person obtaining Signature Date

Consent
