# Supplementary material for: Antenatal couples’ counselling in Uganda (ACCU): study protocol for a randomised controlled feasibility trial
Source: Pilot Feasibility Stud. 2022 Apr 29;8:97. doi: 10.1186/s40814-022-01049-5 (PMC9051788; doi:10.1186/s40814-022-01049-5)
Supplement: Supplementary file 3 — Additional file 3. ACCU data collection tool. [file 40814_2022_1049_MOESM3_ESM.docx]

## **ACCU DATA COLLECTION TOOL**

## **A. ACCU PROJECT_DATA COLLECTION MONTHLY BY HEALTH WORKERS**

Version 2.0

Name of the health facility………………………………………….

Month and year……………………………………………………..

**NB: The questions refers to the services offered in the month.**

| Question | | Response |
| --- | --- | --- |
| 1 | How many women attended ANC at the health facility? |  |
| 2 | How many of these women attended with their partner? |  |
| 3 | How many couples were counselled about long-acting reversible (LARC) methods of post-partum family planning (PPFP) in antenatal clinic? |  |
| 4 | How many couples were offered counselling on both birth planning and use post-partum LARC at the same visit? |  |
| 5 | How many couples in antenatal clinic provided consent to use post-partum LARC in a single session of counselling? |  |
| 6 | How many couples in antenatal clinic provided consent to use post-partum LARC at a subsequent session of counselling? |  |
| 7a | How many women used post-partum family planning within one week of giving birth? |  |
| 7b | Specify method a) Implants |  |
|  | b) IUDs |  |
|  | c) Bilateral tubal ligation |  |
|  | d) Other, specify |  |
| 8a | How many women used post-partum family planning after one week of giving birth? |  |
| 8b | Specify method a) Implants |  |
|  | b) IUDs |  |
|  | c) Bilateral tubal ligation |  |
|  | d) Other, specify |  |
| 9 | How many days was there stock out of implants? |  |
| 10 | How many days was there stock out of IUDs? |  |
| 11 | How many women delivered at the health facility during the last month? |  |
| **For intervention arm only** | | |
| 12 | How many couples attended antenatal clinics and counselling at weekends? |  |
| 13 | On how many days were films on family planning screened in antenatal clinics? |  |
| 14 | How many times were health workers consulted by the VHTs who were doing couples’ counselling? |  |

Thank you

Compiled by……………………………………

## **B. ACCU PROJECT_IN-DEPTH INTERVIEW GUIDE**

**Version 2.0.**

**INTRODUCTION**

*Welcome*

We are interested in your views about the process of our intervention on antenatal couples’ counselling for postpartum family planning and birth planning. This is not a test and you are not being judged. There are no right or wrong answers, .Everything that you say will be kept confidential and your name and any other identifiable information will be taken out of the record.

Opportunity to ask questions and sign consent form

*Read out participant information sheet/get him/her to read it if literate.*

- Do you have any further questions?
- Would you like to take part in the study / go ahead with the interview?
- Do you understand and are you happy with the study information?

*If yes: 1) Read consent form with participant and ask to sign or make a thumb print*

*2) Collect the following quantitative data from the participant*

| **Data form for each participant** | | |
| --- | --- | --- |
| Question | | Response |
| 1 | Age in years |  |
| 2 | - - Religion 1= Catholic, 2=Church of Uganda 3= Seventh Day Adventist 4= Moslem 5= Other, specify |  |
| 3 | - - Highest Level of education attained 1=No formal education, 2= Primary school 3= Secondary school; 4 = Higher education |  |
| 4 | - - Marital/relationship status 1=married/in union, 2=never married 3=separated/divorced, 4= widow 5= Cohabiting |  |
| 5 | Which of the following best describes your main work status over the past 12 months?  1=commercial farmer, 2=trader, 3= salaried employment, 4=house wife, 5=casual labourer 6= Other, specify…….. |  |
| 6 | How many children do you have? |  |
| 7 | Do you want to have another child in the next two years? |  |

- Is it ok to start the interview now?
- I am going to turn on the tape recorder now and we can start.

**1. QUESTIONS FOR VHTs**

**1. Can you tell me what it has been like taking part in this research?**

Prompts: - Can you tell me about what you liked about the study?

-Can you tell me about the things you did not like about the study?

-How did you find engaging the couple to talk?

-How was the reception/ acceptability for your visits?

-How did you find visiting the couple five times at their home and talking/ asking about related topics?

-What was your experience of interference from other members of the extended family? How did you cope with that?

**2. What was your experience of delivering the intervention?**

Prompts:

- Can you tell me about the aspects of delivering the intervention that worked well?
- Can you tell me about the aspects of delivering the intervention that were challenging or didn’t go to plan?
- Can you tell me about any changes in the planned intervention? Why was the change (s) made?

***About family planning***

- How did you feel about discussing options for family planning to use after the delivery with a couple at their home?
- Can you tell me about the things that worked well when discussing options for family planning with the couple at their home?
- Can you tell me about any difficulties you had discussing options for family planning with the couple at their home?

**About birth planning**

- How did you feel about discussing birth planning with the couple at their home?
- Can you tell me about the things that worked well when discussing birth planning with the couple at their home?
- Can you tell me about the things that did not work well when discussing birth planning with the couple at their home?

***About using a smart phone to enter data***

- How did you find using a smart phone to enter data into the Cosmos system?
- Can you tell me about the things that worked well when using a smart phone to enter the data into Cosmos system?
- Can you tell me about anything that did not work well when using a smart phone to enter the data into Cosmos system?
- How did you feel showing the family planning films on the smart phone to the couple?
- How did the couple react when you showed them the films?

**3. Questions about your life/ work.**

**a) What aspects of your life / work helped you to deliver the intervention?**

Prompt: -Can you tell me about anything that helped you to deliver the intervention?

**b) What aspects of your life / work made it difficult to deliver the intervention?**

Prompt: - Can you tell me about any difficulties you had delivering the intervention? What happened?

**4. If we were to involve more areas in this intervention,**

a) What things/factors may make it successful?

b) What things may make it fail?

c) What things should be done differently from what was done here?

**2. QUESTIONS FOR HEALTH SERVICE PROVIDERS**

**1. Can you tell me what it has been like taking part in this study?**

Prompts

- Can you tell me about what you liked about the study?
- Can you tell me about the things you did not like about the study?
- How did you feel engaging couples to talk at the clinic?
- How was the reception/ acceptability of couples attending your facility?

**2. How was your experience in delivering the intervention?**

Prompts:

- Can you tell me about the aspects of delivering the intervention that worked well?
- Can you tell me about the aspects of delivering the intervention that were challenging or did not go to plan?
- Can you tell me about any changes in the planned intervention? Why was/were change (s) made?

***About family planning***

- How did you feel about discussing options for family planning to use after the delivery with couples at the clinic?
- Can you tell me about the things that worked well when discussing options for family planning with couples at the clinic?
- Can you tell me about any difficulties you had discussing options for family planning with couples at the clinic?

***About birth planning***

- How did you feel about discussing birth preparedness and complication readiness with the couple at the clinic?
- Can you tell me about the things that worked well when discussing birth planning with the couple at the clinic?
- Can you tell me about the things that did not work well when discussing birth preparedness and complication readiness with the couple at the clinic?
- How did you feel showing the family planning films in the clinic to the couples?
- How did the couple react when you showed them the films?
- How did the couples react to your messages?

**3. Questions about your life / work.**

**a) What aspects of your life / work helped you to deliver the intervention?**

Prompt: -Can you tell me about anything that helped you to deliver the intervention?

**b) What aspects of your life / work made it difficult to deliver the intervention?**

Prompt: - Can you tell me about any difficulties you had delivering the intervention? What happened?

How did you feel having to work on extra day to offer the intervention?

**4. If we were to involve more areas in this intervention,**

a) What things/factors may make it successful?

b) What things may make it fail?

c) What things should be done differently from what was done here?

**3. QUESTIONS FOR POSTPARTUM WOMEN AND THEIR PARTNERS, POST- PARTUM WOMEN AND MEN**

**1. How was your experience taking part in the study?**

Prompts:

- Please tell me about what you liked about the study?
- Please tell me about the things you did not like about the study?
- How did you feel talking together as a couple about the birth planning and post-partum family planning?
- How did you feel being visited five times by a VHT at your home?
- How did you feel about answering their questions for the survey on the smartphone?

**2. How was your experience in taking part in couples’ counselling with the VHT?**

Prompts:

- How did you feel about being counselled as a couple by a VHT at your home?
- Probe: Did the VHT show you any film(s) and which one(s)?
- If yes - How did you feel being shown the films on the smart phone at your home?
- How did you feel about discussing options for family planning to use after the delivery with the VHT at your home?
- How did you feel about discussing options for place of delivery with the VHT?
  - Probes (for all of the above):
  - What did you like? What did you not like?
  - Can you tell me about anything that helped you to take part in the couples’ counselling with the VHT?
  - Can you tell me about any difficulties you had in taking part in couples’ counselling with the VHT? What happened?
  - How was the involvement of the VHT in the decision making?
  - How was involvement of your partner in decision making?
  - How do you feel about the advice given?
  - Were there any parts of the advice which encouraged you to do birth planning?
  - Were there any parts of the advice which discouraged you from birth planning?
  - Were there any parts of the advice which encouraged you to use family planning after your delivery?
  - Were there any parts of the advice which discouraged you from using family planning after your delivery?

**3. How was your experience in taking part in couples’ counselling at the antenatal clinic?**

- How did you feel about attending the clinic as a couple?
- Probe: Did you watch any film(s) at the clinic and which one(s)?
  - If yes - How did you feel about the films at the clinic?
- How did you feel about discussing options for birth planning with a health worker?
- How did you feel about discussing options for family planning to use after the delivery in the clinic with the health worker?
  - Probes (for all of the above): what did you like? What did you not like?
  - Can you tell me about anything that helped you to take part in the couples’ counselling at the antenatal clinic?
  - Can you tell me about any difficulties you had in taking part in couples’ counselling at the antenatal clinic? What happened?
  - How was the involvement of the health worker in the decision making?
  - How was involvement of your partner in decision making?
  - How do you feel about the advice given?
  - Were there any parts of the advice which encouraged you to plan for your birth and place of delivery?
  - Were there any parts of the advice which discouraged you from planning for your birth and place of delivery?
  - Were there any parts of the advice which encouraged you to use family planning after your delivery?
  - Were there any parts of the advice which discouraged you from using family planning after your delivery?
  - Can you tell me about any difficulties you had in taking part in couples’ counselling at the antenatal clinic? What happened?
- Can you tell me about what you liked about decisions made during counselling?
- Can you tell me about what you did not like about decisions made during counselling?
- Can you tell me about anything you would have liked to be done differently?

**4. Can you tell me about your reasons for taking up or not taking up the offer of a contraceptive method?**

Prompts:

- How do you feel about the offer of a contraceptive method after delivery?

**5. Can you tell me about your reasons for delivering (or not) in the recommended place?**

Prompts:

- How do you feel about the offer of a contraceptive method after delivery?

**6. If we were to involve more areas in this intervention,**

a) What things/factors may make this project successful?

b) What things may make this project fail?

c) What things should be done differently from what was done here?

**4. QUESTIONS FOR CLINIC MANAGERS**

**1. What have been your experiences of taking part in the couples’ counselling study?**

Prompts:

- What went well? Why?
- What was challenging? Why?
- What could be improved?

**2. What has been your experience of PPFP services?**

Prompts

- What went well? Why?
- What was challenging? Why?
- What could be done to overcome these challenges?

**3. If we were to involve more areas in this intervention,**

a) What things/factors may make this project successful?

b) What things may make this project fail?

c) What things should be done differently from what was done here?

**CLOSING COMMENTS**

- *Ask for any additional comments.*
- “Is there anything else you would like to say before we end the interview?”
- *Thank the participant.*
- *Turn recorder off.*

## **C. ACCU PROJECT_FOCUS GROUP DISCUSSION INTERVEIW GUIDE**

**Version 2.0.**

**INTRODUCTION**

*Welcome*

We are interested in your views about the process of our intervention on antenatal couples’ counselling for postpartum family planning and birth planning. This is not a test and you are not being judged. There are no right or wrong answers, .Everything that you say will be kept confidential and your names and any other identifiable information will be taken out of the record. Also we ask you to keep what you hear from this discussion as a secret.

Opportunity to ask questions and sign consent form

*Read out participant information sheet/get them to read it if literate.*

- Do you have any further questions?
- Would you like to take part in the study / go ahead with the interview?
- Do you understand and are you happy with the study information?

*If yes: 1) Read consent form with participant and ask to sign or make a thumb print*

*2) Collect the following quantitative data from each participant*

| **Data form for each participant** | | |
| --- | --- | --- |
| Question | | Response |
| 1 | Age in years |  |
| 2 | - - Religion 1= Catholic, 2=Church of Uganda 3= Seventh Day Adventist 4= Moslem 5= Other, specify |  |
| 3 | - - Highest Level of education attained 1=No formal education, 2= Primary school 3= Secondary school; 4 = Higher education |  |
| 4 | - - Marital/relationship status 1=married/in union, 2=never married 3=separated/divorced, 4= widow 5= Cohabiting |  |
| 5 | Which of the following best describes your main work status over the past 12 months?  1=commercial farmer, 2=trader, 3= salaried employment, 4=house wife, 5=casual labourer 6= Other, specify…….. |  |
| 6 | How many children do you have? |  |
| 7 | Do you want to have another child in the next two years? |  |

- Is it ok to start the interview now?
- I am going to turn on the tape recorder now and we can start.

**1. QUESTIONS FOR VHTs**

**1. Can you tell me what it has been like taking part in this research?**

Prompts: - Can you tell me about what you liked about the study?

-Can you tell me about the things you did not like about the study?

-How did you find engaging the couple to talk?

-How was the reception/ acceptability for your visits?

-How did you find visiting the couple five times at their home and talking/ asking about related topics?

-What was your experience of interference from other members of the extended family? How did you cope with that?

**2. What was your experience of delivering the intervention?**

Prompts:

- Can you tell me about the aspects of delivering the intervention that worked well?
- Can you tell me about the aspects of delivering the intervention that were challenging or didn’t go to plan?
- Can you tell me about any changes in the planned intervention? Why was the change (s) made?

***About family planning***

- How did you feel about discussing options for family planning to use after the delivery with a couple at their home?
- Can you tell me about the things that worked well when discussing options for family planning with the couple at their home?
- Can you tell me about any difficulties you had discussing options for family planning with the couple at their home?

**About birth planning**

- How did you feel about discussing birth planning with the couple at their home?
- Can you tell me about the things that worked well when discussing birth planning with the couple at their home?
- Can you tell me about the things that did not work well when discussing birth planning with the couple at their home?

***About using a smart phone to enter data***

- How did you find using a smart phone to enter data into the Cosmos system?
- Can you tell me about the things that worked well when using a smart phone to enter the data into Cosmos system?
- Can you tell me about anything that did not work well when using a smart phone to enter the data into Cosmos system?
- How did you feel showing the family planning films on the smart phone to the couple?
- How did the couple react when you showed them the films?

**3. Questions about your life/ work.**

**a) What aspects of your life / work helped you to deliver the intervention?**

Prompt: -Can you tell me about anything that helped you to deliver the intervention?

**b) What aspects of your life / work made it difficult to deliver the intervention?**

Prompt: - Can you tell me about any difficulties you had delivering the intervention? What happened?

**4. If we were to involve more areas in this intervention,**

a) What things/factors may make it successful?

b) What things may make it fail?

c) What things should be done differently from what was done here?

**2. QUESTIONS FOR POSTPARTUM WOMEN AND THEIR PARTNERS, POST- PARTUM WOMEN AND MEN**

**1. How was your experience taking part in the study?**

Prompts:

- Please tell me about what you liked about the study?
- Please tell me about the things you did not like about the study?
- How did you feel talking together as a couple about the birth planning and post-partum family planning?
- How did you feel being visited five times by a VHT at your home?
- How did you feel about answering their questions for the survey on the smartphone?

**2. How was your experience in taking part in couples’ counselling with the VHT?**

Prompts:

- How did you feel about being counselled as a couple by a VHT at your home?
- Probe: Did the VHT show you any film(s) and which one(s)?
- If yes - How did you feel being shown the films on the smart phone at your home?
- How did you feel about discussing options for family planning to use after the delivery with the VHT at your home?
- How did you feel about discussing options for place of delivery with the VHT?
  - Probes (for all of the above):
  - What did you like? What did you not like?
  - Can you tell me about anything that helped you to take part in the couples’ counselling with the VHT?
  - Can you tell me about any difficulties you had in taking part in couples’ counselling with the VHT? What happened?
  - How was the involvement of the VHT in the decision making?
  - How was involvement of your partner in decision making?
  - How do you feel about the advice given?
  - Were there any parts of the advice which encouraged you to do birth planning?
  - Were there any parts of the advice which discouraged you from birth planning?
  - Were there any parts of the advice which encouraged you to use family planning after your delivery?
  - Were there any parts of the advice which discouraged you from using family planning after your delivery?

**3. How was your experience in taking part in couples’ counselling at the antenatal clinic?**

- How did you feel about attending the clinic as a couple?
- Probe: Did you watch any film(s) at the clinic and which one(s)?
  - If yes - How did you feel about the films at the clinic?
- How did you feel about discussing options for birth planning with a health worker?
- How did you feel about discussing options for family planning to use after the delivery in the clinic with the health worker?
  - Probes (for all of the above): what did you like? What did you not like?
  - Can you tell me about anything that helped you to take part in the couples’ counselling at the antenatal clinic?
  - Can you tell me about any difficulties you had in taking part in couples’ counselling at the antenatal clinic? What happened?
  - How was the involvement of the health worker in the decision making?
  - How was involvement of your partner in decision making?
  - How do you feel about the advice given?
  - Were there any parts of the advice which encouraged you to plan for your birth and place of delivery?
  - Were there any parts of the advice which discouraged you from planning for your birth and place of delivery?
  - Were there any parts of the advice which encouraged you to use family planning after your delivery?
  - Were there any parts of the advice which discouraged you from using family planning after your delivery?
  - Can you tell me about any difficulties you had in taking part in couples’ counselling at the antenatal clinic? What happened?
- Can you tell me about what you liked about decisions made during counselling?
- Can you tell me about what you did not like about decisions made during counselling?
- Can you tell me about anything you would have liked to be done differently?

**4. Can you tell me about your reasons for taking up or not taking up the offer of a contraceptive method?**

Prompts:

- How do you feel about the offer of a contraceptive method after delivery?

**5. Can you tell me about your reasons for delivering (or not) in the recommended place?**

Prompts:

- How do you feel about the offer of a contraceptive method after delivery?

**6. If we were to involve more areas in this intervention,**

a) What things/factors may make this project successful?

b) What things may make this project fail?

c) What things should be done differently from what was done here?

**CLOSING COMMENTS**

- *Ask for any additional comments.*
- “Is there anything else you would like to say before we end the interview?”
- *Thank the participants.*
- *Turn recorder off.*

## **D. QUESTIONNAIRES USED BY VHTS**

**Version 2.0**

*These will be put onto COSMOS app. The VHTs will be asked to get this information from the pregnant women and their partners and children.*

Participant ID will be a number from

- - RU = Rubaya 000-0400
  - KG = Kagongi 0401-0800
  - BI = Biharwe 0801-1200
  - KK = Kakoba 1201- 1600

### **D 1. BASELINE ANTENATAL VISIT**

| **INFORMATION RECORDED AT EACH VISIT** | | |
| --- | --- | --- |
|  | Item | Response |
| 1 | VHT code | __/___/ |
| 2 | Participant ID | __/___/___/ |
| 3 | Date of visit | Day/month/year |

| **CHECKLIST OF INCLUSION CRITERIA: include only if the participant answers NO to questions4-6 and YES to questions 7-10.** | | | |
| --- | --- | --- | --- |
| Question | | Response | |
|  |  | YES | NO |
| 4 | Is the pregnancy more than seven months (self-report)? |  |  |
| 5 | Does the woman have any severe medical/physical condition(s) making her unable to answer the questions? |  |  |
| 6 | Does the woman have any condition (such as mental illness) which makes her unable to give informed consent? |  |  |
| 7 | Is the woman a resident of this area? |  |  |
| 8 | - Do you plan to attend Antenatal clinic and postnatal clinic in the study health facility… (mention your health center III)? |  |  |
| 9a | - Is the pregnant woman in a relationship with the father of their expected child? |  |  |
| 9b | - If yes, have they been together in a relationship for at least 6 months? |  |  |
| 10 | - Does the woman consent to participate in this study? |  |  |

| **DEMOGRAPHICS** | | |
| --- | --- | --- |
| **PREGNANT WOMAN** | | |
| Question | | Response |
| 11 | Age in years |  |
| 12 | - - Religion 1= Catholic, 2=Church of Uganda 3= Seventh Day Adventist 4= Moslem 5= Other, specify |  |
| 13 | - - Highest Level of education attained 1=No formal education, 2= Primary school 3= Secondary school; 4 = Higher education |  |
| 14 | - - Marital/relationship status 1=married/in union, 2= Cohabiting 3=separated/divorced, 4= widow |  |
| 15 | Which of the following best describes your main work status over the past 12 months?  1=commercial farmer, 2=trader, 3= salaried employment, 4=house wife, 5=casual labourer 6= other, specify |  |
| **PARTNER / HUSBAND** | |  |
| 16 | - - Age |  |
| 17 | - - Religion; 1= Catholic, 2=Church of Uganda 3= Seventh Day Adventist 4= Moslem 5= Other, specify |  |
| 18 | Level of education; 1=No formal education, 2= Primary school 3= Higher than primary |  |
| 19 | Which of the following best describes your main work status over the past 12 months? 1=commercial farmer, 2=trader, 3= salaried employee, 4=casual labourer, 5= other (specify) |  |

| **PAST MEDICAL HISTORY FOR PREGNANT WOMAN** | | | |
| --- | --- | --- | --- |
| Question | | Response | |
|  |  | YES | NO |
| 20 | - - - Have you ever been told by a doctor or other health worker that you have HIV/AIDS? |  |  |
| 21 | - - - Have you ever been told by a doctor or other health worker that you have TB (Tuberculosis)? |  |  |
| 22 | Have you ever been told by a doctor or other health worker that you have raised blood sugar or diabetes? |  |  |
| 23 | Have you ever been told by a doctor or other health worker that you have raised blood pressure or hypertension? |  |  |
| 24 | - - - Have you ever been told by a doctor or other health worker that you have Sickle cell disease? |  |  |
| 25 | - - - Have you ever been told by a doctor or other health worker that you have heart disease? |  |  |
| 26 | - - - Have you ever been told by a doctor or other health worker that you have kidney disease? |  |  |
| 27a | - - - Have you ever been operated on the uterus such as a Caesarean section? |  |  |
| 27b | - - - If yes, how many times? |  |  |
| 28 | - - - A) Have you ever been told by a doctor or other health worker that you have any other serious medical condition?     - B) Specify: … |  |  |

| **OBSTETRIC HISTORY** | | |
| --- | --- | --- |
| **PREVIOUS HISTORY** | | |
| Question | | Response |
| 29 | How many previous pregnancies have you had before this one? … [number]  **If this is the first pregnancy SKIP to question 42** |  |
| 30 | How many live girls do you have? Number… |  |
| 31 | How many live boys do you have? Number… |  |
| 32 | Are all the children from the same biological father? Yes/No |  |
| 33 | How old were you when your (first) child was born? |  |
| 34 | Have you ever lost a baby or child? Yes /No  Yes/No **If no skip to qn 36** |  |
| 35 | If yes – how many?   - Stillbirths - Deaths of a baby aged less than 1 month old |  |
|  | -Deaths of children more than 1 month old |  |
| 36 | Have you ever had any serious problems from a delivery? Specify all that apply: |  |
|  | Difficult or prolonged labour (more than 24 hours)? |  |
|  | Ruptured uterus |  |
|  | Tear of vaginal wall |  |
|  | Heavy bleeding after the delivery? |  |
|  | Other , specify |  |
| **About past pregnancy** | |  |
| 37 | - - Year of delivery |  |
| 38 | - - Place of delivery 1= Health facility 2= Home 3= Other places (specify) |  |
| 39 | If health facility, specify the level  1= HCII 2= HCIII 3=HCIV 4. Government general hospital 5=Referral hospital 6=Private clinic 7= Private general hospital |  |
| 40 | - - Type of delivery: 1=Normal Vaginal Delivery 2=Caesarean Section 3=Instrumental delivery |  |
| 41 | - - Was the pregnancy 1= single 2= Multiple pregnancy |  |
| **About Current Pregnancy** | |  |
| 42 | Was this pregnancy conceived after 1=Trying for pregnancy 2=Not trying, not using contraceptive  3=Not trying, using contraceptive |  |
| 43 | - What was the date of your last menstrual period? (please estimate)   If you do not know, how many months have you been pregnant? |  |
| 44a | Have you already attended Antenatal clinic (ANC)? Yes/No |  |
| 44b | If yes, how many times? |  |
| 45a | - - Did your husband/partner ever go with you? Yes/No |  |
| 45b | - - Who made the final decision to attend antenatal clinic together or not?   - 1= Woman alone 2= Male partner alone   - 3= Joint decision of the couple   - 4= woman with another person apart from male partner |  |
| 45c | - - Name of health centre where you attend Antenatal clinic |  |
| 46a | - - Have you already received counselling/ discussed about/ talked about     - Use of family planning contraceptive after delivery? Yes/No |  |
| 46b | Have you already received counselling/ discussed about/ talked about where you are planning to deliver from and other preparations for the birth? Yes/No |  |
| - - - 47. Have you experienced any of the following during this pregnancy | |  |
| 47a | - - Heavy bleeding during pregnancy? Yes/No |  |
| 47b | - - Multiple pregnancy? (twins or triplets) Yes/No/Not sure |  |
| 47c | - - Baby is not in the right position? Yes/No |  |
| 48 | Do you intend to conceive again after this pregnancy Yes/ No  **If NO, skip to qn 50** |  |
| 49 | If you intend to conceive again, how long after you deliver? |  |
| 50 | 1. Do you intend to use Family Planning after this pregnancy? 1=Yes/ 2=No 3= Don’t Know/not sure 2. **If NO or not sure skip to qn 53** |  |
| 51 | - 1. If yes, which type? 1=Implant 2=IUD 3= Either Implant or IUD, 4= Others, specify |  |
| 52 | How long after delivery? 1=Immediately (within 2 days) 2==Around 6 weeks; 3 = 6 weeks – 6 months; 4: 6-12 months |  |
| 53 | - - 1. Who will make the final decision about using family planning?   1= Woman alone 2= Male partner alone 3= Joint decision of the couple 4= woman with another person apart from male partner |  |
| **About where to give birth** | |  |
| 54 a | Have you already decided where you will give birth? Yes/No |  |
| 54 b | If yes, where? Home / Health Centre II / Health Centre III / Health Centre IV / Hospital |  |
| 54c | If a health centre or hospital, Specify name:… |  |
| 54d | Who made the final decision where to deliver?  1= Woman alone 2= Male partner alone 3= Joint decision of the couple 4= woman with another person apart from male partner |  |
| **For woman only and in privacy** | |  |
| 55 | Have you ever used a family planning method? Yes/No  **If no skip to qn 57** |  |
| 56 | If Yes, which type(s)? [tick all that apply]  1=Pills, |  |
|  | 2=condoms |  |
|  | 3=injectable |  |
|  | 4=Implants |  |
|  | 5=IUD |  |
|  | 6=Other, specify |  |
| 57 | After giving birth, would you like to use family planning in secret without the knowledge of your spouse? Yes /No  If No, skip to Q 59 |  |
| 58 | If yes, which method would you want to use 1= Implant 2= IUD 3= Either Implant or IUD 4= other specify |  |
| 59 | Has any one hit, slapped, kicked, or done anything else to hurt you physically in the last 6 months? Yes / No  If no, END. |  |
| 60 | If yes, who has done any of these things to physically hurt you in the last 6 months?  1 = Current husband / partner; 2 = Other, specify  [*Please counsel and support the woman to disclose this to the focal person for gender-based violence at the health facility, or the Local Council I leader (LCI)]* |  |

| **QUESTIONS FOR VHT** | | |
| --- | --- | --- |
| Question | | Response |
| 59a | Was the woman’s husband / partner present at this visit? Yes / No  **If No, skip to qn 60** |  |
| 59b | If Yes, did he consent to take part in the project? Yes / No  **If Yes , skip to qn 60a** |  |
| 59c | If no, why not? (free text) |  |
| 60a | Did you do counselling at this visit: Yes/No  **If no Skip to question 65** |  |
| 61 | - - Who was counselled?     - 1=Woman only 2=Woman and her partner together 3=Partner only 4=Other family member/friend |  |
| 62Among other topics did you counsel/ advise about;(tick all that apply) | |  |
| 62a | - - - HIV testing Yes/No |  |
| 62b | - - - Use of family planning after giving birth Yes/No |  |
| 62c | - - - Planning where to deliver from and other preparations for the birth? Yes/ No |  |
| 62d | Benefits of attending ANC as a couple Yes/No |  |
| 63 | For Intervention area only: Was Drama film shown at this visit? Yes/No |  |
| 64 | For Intervention area only: Was Documentary film shown at this visit? Yes/No |  |
| 65 | Why was counselling not done during this visit?  1=Woman refused / did not have time 2=Partner refused / did not have time 3=VHT did not have time 4=Other (please specify) |  |
| 66 | Any other comment about this visit |  |

## **D 2. FOLLOW-UP ANTENATAL VISIT**

(wait at least 4 weeks after baseline visit)

| **INFORMATION RECORDED AT EACH VISIT** | | |
| --- | --- | --- |
|  | Item | Response |
| 1 | VHT code | __/___/ |
| 2 | Participant ID | __/___/___/ |
| 3 | Date of visit | Day/month/year |
| 4 | Is the partner present during this meeting | Yes / no |

| **Intentions about birth planning and family planning** | | |
| --- | --- | --- |
| 5a | - - Since the last study visit, did you agree on a place of delivery? Yes/No; If NO, Skip to question 6 |  |
| 5b | - - If Yes, a) where? Name the place |  |
| 5c | - - If yes, b) level of care. 1= Home, 2= TBA 3= HC11, 4=HC111, 5=HCIV 6= Government General Hospital 7= Referral hospital 8= Private clinic/maternity home 9= Private general hospital |  |
| 5d | b) Who made the final decision?  1= Woman alone 2= Male partner alone 3= Joint decision of the couple 4= woman with another person apart from male partner |  |
| 6a | - - - - Have you decided whether to use family planning after delivery? Yes/No. **If NO, Skip to question 7a1** |  |
| 6b | - - - - If yes, which type? (options)1=Implant 2=IUD 3= Either implant or IUD 4=Others specify |  |
| 6c | - Would you like to receive the chosen method of contraception before discharge from the health facility centre? Yes/No, Not sure |  |
| 6d | - Who made the final decision?   1= Woman alone 2= Male partner alone 3= Joint decision of the couple 4= woman with another person apart from male partner |  |
| **Antenatal clinic attendance** | | |
| 7a1 | Have you attended Antenatal Clinic since the baseline (study) visit? Yes/No **If NO, skip to question 19** |  |
| 7a2 | If yes, how many times since baseline visit? |  |
| **For qn 7b and 8, repeat for each visit made** | | |
| 7b | On what day of the week did you attend? Weekday / weekend |  |
| 8a | - If you attended ANC: Did your partner accompany you? Yes/ No |  |
| 8b | - If no, why not? - 1= lack of time; 2 = lack of money for transport; 3= Does not feel it is important; 4 = Feels it is shameful; 5 = Other, specify |  |
| 9a | Who made the final decision whether the partner accompanied you or not?  1= Woman alone, 2= Male partner alone, 3= Joint decision of the couple, 4= woman with another person apart from male partner |  |
| 9b | - - Did you need to spend any money in order to get to and from the ANC? Yes / No |  |
| 9c | - - If Yes, how much did you spend? |  |
| 9d | - - Did you need to pay anything else for the ANC? Yes/no |  |
| 9e | - - If yes, how much? |  |
| 9f | - - And for what? |  |
| 9g | - - Did the woman lose any earnings because of time taken to attend ANC?   - – If yes, how much? |  |
| 9h | - - If the partner attended, did he lose any earnings because of the time taken?   - If yes, how much? |  |
| - - **Birth planning and complication readiness while at health facility: Did you receive counselling on/ discussion about the following…** | | |
| 10a | - - - - With whom do you plan to go for delivery? Yes/ No |  |
| 10b | - - - - If **QN 10a** is yes, whom will you go with? 1=partner 2=mother 3=sister 4=friend 5= other, specify … |  |
| 11 | - - - - Who will stay at home to take care of the home while you are away? Yes/No |  |
| 12a | Did you discuss whether you are at risk of any problems during pregnancy/delivery? Yes/No |  |
| 12b | - - - - Did the health worker tell you if your pregnancy was ok? Yes No |  |
| 12c | Did the health worker advise you where it would be best for you to deliver your baby? Yes / No |  |
| 12d | - - - - If yes, where did they advise? |  |
| 13 | - - - - Arrangement for transport? Yes /No |  |
| 14 | - - - - Saving money for delivery or emergency? Yes / No |  |
| 15 | - - - - Identifying skilled birth attendant? Yes / No |  |
| 16 | - - - - Buying some supplies needed during delivery? Yes/ No |  |
| **Family planning counselling and intention to use** | | |
| 17a | - - - - Did you see the d**ocumentary** film on family planning? Yes/No |  |
| 17b | - - - - Did you see the **drama** film on family planning? Yes/No |  |
| 18 | - - - - Were you counselled about/ did you discuss about use of family planning after giving birth during last Antenatal clinic visit? Yes/ No. |  |
|  | - **Non-attendance at ANC** |  |
| 19 | - Why did you not attend? |  |
| 20 | - - - - Are you planning to attend? Yes /No |  |
|  | - - - - **For woman only and in privacy** |  |
| 21 | Are you interested in using family planning after delivery? Yes/No |  |
| 22 | If yes, which type? 1= Implant 2=IUD 3= Either implant or IUD 4=Others specify |  |
| 23 | Would you use family planning after giving birth in secret without the knowledge of your spouse? Yes /No **If No, Skip to Q 25** |  |
| 24 | If yes, which method would you want to use 1= Implant 2= IUD 3= Either Implant or IUD 4= other specify |  |
| 25 | Since my last visit to you, has any one hit, slapped, kicked, or done anything else to hurt you physically? Yes / No **If No, END**. |  |
| 26 | If yes, were you seriously injured (needing to go to hospital)? Yes / No  [If yes, complete serious adverse event form] |  |
| 27 | If yes, who has done any of these things to physically hurt you?  1 = Current husband / partner; 2 = Other, specify  [*Please counsel and support the woman to disclose this to the focal person for gender-based violence at the health facility, or the Local Council I leader (LCI)]* |  |
| 28 | If current husband / partner, was this related to a discussion about birth planning or family planning? Yes / No |  |
| **For man only and in privacy** | |  |
| 29 | Since my last visit to you, has any one hit, slapped, kicked, or done anything else to hurt you physically? Yes / No **If No, END**. |  |
| 30 | If yes, were you seriously injured (needing to go to hospital)? Yes / No  [If yes, complete serious adverse event form] |  |
| 31 | If yes, who has done any of these things to physically hurt you?  1 = Current wife / partner; 2 = Other, specify  [*Please counsel and support the man to disclose this to the focal person for gender-based violence at the health facility, or the Local Council I leader (LCI)]* |  |
| 32 | If current wife / partner, was this related to a discussion about birth planning or family planning? Yes / No |  |

| **QUESTIONS FOR VHT** | | |
| --- | --- | --- |
| Question | | Response |
| 33a | Was the woman’s husband / partner present at this visit? Yes / No |  |
| 33 b | Did you do counselling at this visit: Yes/No  **If no Skip to question 30** |  |
| 34 | - - Who was counselled?     - 1=Woman only 2=Woman and her partner together 3=Partner only 4=Other family member/friend |  |
| 35Among other topics did you counsel/ advise about;(tick all that apply) | |  |
| 35a | - - - HIV testing Yes/No |  |
| 35b | - - - Use of family planning after giving birth Yes/No |  |
| 35c | - - - Birth planning and complication readiness Yes/ No |  |
| 35d | Benefits of attending ANC as a couple Yes/No |  |
| 36 | Was Drama film shown at this visit? Yes/No |  |
| 37 | Was Documentary film shown at this visit? Yes/No |  |
| 38 | Why was counselling not done during this visit? |  |
| 39 | Any other comment about this visit |  |

### **D 3. POST-PARTUM VISIT 1 (BY DAY 7)**

| **INFORMATION RECORDED AT EACH VISIT** | | |
| --- | --- | --- |
|  | Item | Response |
| 1 | VHT code | __/___/ |
| 2 | Participant ID | __/___/___/ |
| 3 | Date of visit | Day/month/year |
| 4 | Is the partner present during this meeting? Yes/No |  |

| **QUESTIONS ABOUT ANTENATAL** | | |
| --- | --- | --- |
| 5a | - Did you attend ANC since our last (study) visit? Yes/No - **If No skip to qn 7** |  |
| 5b | - If yes, how many times? (since the last study visit) |  |
| **For qn 5c and 6, please repeat for each visit** | | |
| 5c | - On what day of the week did you attend? Weekday / weekend |  |
| 6a | - If Yes, Did your partner accompany you? Yes/ No |  |
| 6b | - If no, why not? - 1= lack of time; 2 = lack of money for transport; 3= Does not feel it is important; 4 = Feels it is shameful; 5 = Other,specify |  |
| 6c | Who made the final decision whether the partner accompanied you or not?  1= Woman alone 2= Male partner alone 3= Joint decision of the couple 4= woman with another person apart from male partner |  |
| 6d | - - Did you need to spend any money in order to get to and from the ANC? Yes / No |  |
| 6e | - - If Yes, how much did you spend? |  |
| 6f | - - Did you need to pay anything else for the ANC? Yes/No |  |
| 6g | - - If yes, for what? |  |
| 6h | - - How much? |  |
| 6i | - - Did the woman lose any earnings because of time taken to attend ANC?   - – If yes, how much? |  |
| 6j | - - If the partner attended, did he lose any earnings because of the time taken?   – If yes, how much? |  |
| 7 | How many times in total did you attend Antenatal clinic? enter the number e.g 1, 2 |  |
| 8 | How many times did partner accompany you? enter the number e.g 1, 2 |  |
| 9a | For intervention area only: Did you see the **documentary** film about family planning use after giving birth? Yes/No |  |
| 9b | For intervention area only: Did you see the **drama** film about family planning use after giving birth? Yes/No |  |
| **During Antenatal clinic, did you receive counselling on / discuss about/ talk about** | | |
| 10a | The best place for you to deliver? Yes / No |  |
| 10b | 1. If yes, where were you advised to deliver? |  |
| 10c | Use of family planning after delivery? Yes/No **No skip to qn 13** |  |
| 11 | If Yes, did you agree to use it? Yes/No **If No skip to qn 13** |  |
| 12 | If yes, which method did you choose (during pregnancy)? 1= implant, 2= IUD, 3=Sterilisation 4= Pills 5= Injectable 6= Other, specify |  |
| 13a | Were you counselled / advised about HIV testing? Yes/No.  **If NO skip to qn14a** |  |
| 13b | - - - - If yes, did you test? Yes/No |  |
| 13c | Did your partner test? Yes/ No |  |
| 14a | Out of a scale of 10, how were you (woman) satisfied with care received during antenatal? 1 = very dissatisfied; 10 = very satisfied) |  |
| 14b | Out of a scale of 10, how were you (woman) satisfied with care received during delivery? 1 = very dissatisfied; 10 = very satisfied) |  |
| 15a | Out of a scale of 10, how were you (man) satisfied with care given to your wife during antenatal? 1 = very dissatisfied; 10 = very satisfied) |  |
| 15b | Out of a scale of 10, how were you (man) satisfied with care given to your wife during delivery? 1 = very dissatisfied; 10 = very satisfied) |  |

| **DURING DELIVERY** | | |
| --- | --- | --- |
| Question | | Response |
| 16 | Date of delivery (DD/MM/Year) |  |
| 17 | - - Months of pregnancy at time of delivery? |  |
| 18 | - - How long before delivery did your waters break? 1=Less than1 day 2= More than 1 day. **If less than 1 day, skip to question 20** |  |
| 19 | - - - If >1 day, how many days before delivery? |  |
| 20a) | - - Place of delivery (enter the name and level of facility) |  |
| 20b | - - Level of care of place of delivery above. 1= Home, 2= TBA 3= HC11, 4=HC111, 5=HCIV 6= Government General Hospital 7= Referral hospital 8= Private clinic/maternity home 9= Private general hospital |  |
| 20c | The level of birth attendant: 1=none /2=family member 3=TBA 4=nursing assistant 5=nurse / midwife 6= clinical officer 7= General doctor 8= Specialist doctor |  |
| 21a | - - Who made the final decision about the place of delivery?   1= Woman alone 2= Male partner alone 3= Joint decision of the couple 4= woman with another person apart from male partner |  |
| 21b | Was this the same as the place recommended during antenatal clinic?  Yes / No |  |
| 21c | If no, why did you not go to the recommended place? |  |
| 22a | Type of delivery: 1=Normal Vaginal Delivery 2=Caesarean Section 3=instrumental delivery |  |
| 22b | Was this pregnancy a single or multiple 1= single pregnancy 2= multiple pregnancy |  |
| 23a | - Outcome of delivery for mother:1= alive and well 2= maternal death 3=complications (specify) |  |
| 23b1 | - - Outcome of delivery for First baby: 1=alive and well 2=stillbirth 3=neonatal death |  |
| 23b2 | - - Outcome of delivery for Second baby: 1=alive and well 2=stillbirth 3=neonatal death |  |
| 23c1 | - - In case of neonatal death, what is the possible cause of death? |  |
| 23c2 | - - How old was baby at the time of death? |  |
| 24a | - - - Sex of first baby 1= Male 2=Female |  |
| 24b | - - - Sex of the second baby in case of twins: 1= Male 2=Female |  |
| 24c | - - Did you need to spend any money in order to get to and from the place for delivery? Yes / No |  |
| 24d | If Yes, how much did you spend? |  |
| 24e | - - Did you need to pay anything else for the delivery? Yes/no |  |
| 24f | - - If yes, for what? |  |
| 24g | - - How much? |  |

| **Family planning** | | |
| --- | --- | --- |
| Question | | Response |
| 25 | Do you desire another pregnancy within 2 years from now?  1=Yes, 2=No 3=Do not know |  |
| 26a | - Have you had sexual intercourse since delivery Yes/ No |  |
| 26b | Are you exclusively breast feeding the baby? Yes/No |  |
| 27 | - Did you receive any counselling on/ discuss / talk about family planning use after delivery, **when you were in the** **health centre** for delivery? Yes/No |  |
| 28 | - Are you using postpartum contraception? Yes/No - **If No, skip to qn 30** |  |
| 29a | - - If Yes, specify type: 1= implant, 2= IUD, 3=Sterilisation 4= Pills 5= Injectable 6= Other, specify |  |
| 29b | - - If yes, how many days after delivery did you receive the contraceptive? |  |
| 29c | - - Did you need to spend any money in order to get to receive the postpartum contraception? Yes / No |  |
| 29d | - - If yes, how much did you spend? **Skip to qn 31** |  |
| 30 | Why are you not using family planning? 1=baby is still young and hence I am not at risk, 2= I fear family planning will adversely affect the quality of breast milk, 3= I fear the side effects of family planning to me 4= the service I wanted was not available at the place of delivery 5= The service provider to give me the method was not available 6= other, specify |  |
| 31 | - Who made the final decision whether or not to use family planning?   1= Woman alone 2= Male partner alone 3= Joint decision of the couple 4= woman with another person apart from male partner |  |

| **COMPLICATION READINESS** | | |
| --- | --- | --- |
| **Instruction: Ask the respondent to tell you the danger signs/complications which she was aware could have occurred during pregnancy , please tick all the responses mentioned spontaneously** | | |
| 32 | What are the Key obstetric danger signs during pregnancy? | |
|  | Vaginal bleeding |  |
|  | Fits of pregnancy |  |
|  | Swelling of face/swollen hands |  |
|  | Blurred vision |  |
|  | High grade fever |  |
|  | Loss of consciousness |  |
|  | Severe headache |  |
| 33 | What are key obstetric danger signs during childbirth? | |
|  | Severe vaginal bleeding |  |
|  | Prolonged labour (>24hours), |  |
|  | Convulsions |  |
|  | Severe abdominal pain |  |
|  | Retained placenta |  |
| 34 | What are key danger signs during post-partum (after child birth)? | |
|  | severe vaginal bleeding |  |
|  | high fever |  |
|  | foul-smelling vaginal discharge |  |
| **Complications experienced during pregnancy** | |  |
| 35a | Ask if the mother experienced any of the above complications 1=yes 2=N0 |  |
| 35 b | If yes, what did she do? |  |

| **BIRTH PREPAREDNESS DURING PREGNANCY** | | |
| --- | --- | --- |
| Question | | Response |
| 36a | Had you planned where you wanted to deliver? Yes / No |  |
| 36b | Is this the same place where you actually delivered? Yes/No |  |
| 37 | Had you saved money specifically for obstetric emergencies? Yes/ No |  |
| 38 | Had you identified and arranged for means of transport to take you to the facility for emergency or birth? Yes/No |  |
| 39 | Had you prepared delivery kit/ materials to use e.g razor blade to cut the cord? Yes /No |  |
| 40 | Had you arranged with someone to go with you to the health facility at time of delivery? Yes/No |  |
| 41 | Had you made an arranged with someone to look after your home while you are at the facility to deliver? Yes/No |  |

|  | **PRIVATE QUESTIONS TO WOMAN** |  |
| --- | --- | --- |
| 42 | Are you using Family planning in secret without the knowledge of spouse? Yes /No **If No skip to qn 44** |  |
| 43 | If yes, Which method are you using? 1= Implant 2= IUD 3= other specify **End** |  |
| 44 | Would you like to use Family planning in secret without the knowledge of spouse? Yes /No **If No,skip to Q46** |  |
| 45 | Which method would she like to use? 1= Implant 2= IUD 3= other specify |  |
| 46 | Since my last visit to you, has any one hit, slapped, kicked, or done anything else to hurt you physically? Yes / No **If no, END.** |  |
| 47 | If yes, were you seriously injured (needing to go to hospital)? Yes / No  [If yes, complete serious adverse event form] |  |
| 48 | If yes, who has done any of these things to physically hurt you?  1 = Current husband / partner; 2 = Other, specify  [*Please counsel and support the woman to disclose this to the focal person for gender-based violence at the health facility, or the Local Council I leader (LCI)]* |  |
| 49 | If current husband / partner, was this related to a discussion about birth planning or family planning? Yes / No |  |
| **For man only and in privacy** | | |
| 50 | Since my last visit to you, has any one hit, slapped, kicked, or done anything else to hurt you physically? Yes / No **If No, END.** |  |
| 51 | If yes, were you seriously injured (needing to go to hospital)? Yes / No  [If yes, complete serious adverse event form] |  |
| 52 | If yes, who has done any of these things to physically hurt you?  1 = Current wife / partner; 2 = Other, specify  [*Please counsel and support the man to disclose this to the focal person for gender-based violence at the health facility, or the Local Council I leader (LCI)]* |  |
| 53 | If current wife / partner, was this related to a discussion about birth planning or family planning? Yes / No |  |

| **QUESTIONS FOR VHT** | | |
| --- | --- | --- |
| Question | | Response |
| 54a | Was the woman’s husband / partner present at this visit? Yes / No |  |
| 54b | Did you do counselling at this visit: Yes/No **If No Skip to question 59** |  |
| 55 | - - Who was counselled?     - 1=Woman only 2=Woman and her partner together 3=Partner only 4=Other family member/friend |  |
| 56Among other topics did you counsel/ advise about | |  |
|  | - - - Use of family planning after giving birth Yes/No |  |
| 57 | For intervention areas only: Was Drama film shown at this visit? Yes/No |  |
| 58 | For intervention areas only: Was Documentary film shown at this visit? Yes/No |  |
| 59 | Why was counselling not done during this visit? |  |
| 60 | Any other comment about this visit |  |

### **D 4. POSTPARTUM VISIT 2 (6 MONTHS)**

| **INFORMATION RECORDED AT EACH VISIT** | | |
| --- | --- | --- |
|  | Item | Response |
| 1 | VHT code | __/___/ |
| 2 | Participant ID | __/___/___/ |
| 3 | Date of visit | Day/month/year |
| 4 | Is the partner present during this meeting? Yes/No |  |

| Question | | Response |
| --- | --- | --- |
| 5a | - Is the baby still alive? Yes/No |  |
| 5b | - In case of twins; is the second baby still alive? Yes/No |  |
| 5c | - How old was the baby when s/he died? |  |
| 5e | - What is the possible cause of death? |  |
| 6 | - After giving birth, have you received counselling about/ advice about use family planning from health workers? Yes/No |  |
| 7a | - Have your monthly periods resumed? Yes/No |  |
| 7b | Are you breast feeding the baby? Yes/No **If No skip to qn 8** |  |
| 7c | If you are still breastfeeding, Have added other feeds (drinks /solid food for the baby? Yes/No |  |
| 8 | - Have you had sexual intercourse since delivery Yes/ No |  |
| 9 | - Are you pregnant now? Yes/No |  |
| 10 | Do you desire for another pregnancy within 2 years from now?  1=Yes 2=No 3=Do not know |  |
| 11 | - If you had used immediate family planning, are you still having it? Yes/No/ Not applicable   **If yes, skip to qn 17; If not applicable skip to qn 13** |  |
| 12 | If no, why? |  |
| 13 | - Since the last time of my visit to you, Have you started using any family planning method to delay or avoid getting pregnant, Yes/No - **If No, Skip to qn 21** |  |
| 14 | - If yes, when did you start? (Number of weeks after giving birth)… |  |
| 15 | - What type of family planning are you using?   1= implant, 2= IUD, 3=Sterilisation 4= Pills 5= Injectable 6= Other, specify |  |
| 16a | - Who made the final decision about using it? 1= Woman alone 2= Male partner alone 3= Joint decision of the couple 4= woman with another person apart from male partner |  |
| 16b | - - Did you need to spend any money in order to get the family planning? Yes / No |  |
| 16c | - - If Yes, how much did you spend? |  |
| 17 | - Have you experienced any problems / side-effects? Yes/No - **If No, End** |  |
| 18 | - If yes, did you go to the health facility about this? Yes/No |  |
| 19 | - - If yes, did you receive treatment? |  |
| 20a | - - Did the treatment help? Yes/No |  |
| 20b | - - Did you need to spend any money to treat the side-effects? Yes / No |  |
| 20c | - - If Yes, how much did you spend? |  |

| **PRIVATE QUESTIONS TO WOMAN** | | |  |
| --- | --- | --- | --- |
| 21 | Are you using Family planning in secret without the knowledge of spouse? Yes /No **If No skip to qn 23** |  |  |
| 22 | If yes, Which method are you using? 1= Implant 2= IUD 3= other (specify) **Skip to qn25** |  |  |
| 23 | Would you like to use Family planning in secret without the knowledge of spouse? Yes /No **If No, Skip to 25** |  |  |
| 24 | Which method would you like to use? 1= Implant 2= IUD 3= other **specify** |  |  |
| 25 | Since my last visit to you, has any one hit, slapped, kicked, or done anything else to hurt you physically? Yes / No **If No, END**. |  | |
| 26 | If yes, were you seriously injured (needing to go to hospital)? Yes / No  [If yes, complete serious adverse event form] |  | |
| 27 | If yes, who has done any of these things to physically hurt you?  1 = Current husband / partner; 2 = Other, specify  [*Please counsel and support the woman to disclose this to the focal person for gender-based violence at the health facility, or the Local Council I leader (LCI)]* |  | |
| 28 | If current husband / partner, was this related to a discussion about family planning? Yes / No |  | |
| **For man only and in privacy** | | | |
| 29 | Since my last visit to you, has any one hit, slapped, kicked, or done anything else to hurt you physically? Yes / No **If No, END**. |  | |
| 30 | If yes, were you seriously injured (needing to go to hospital)? Yes / No  [If yes, complete serious adverse event form] |  | |
| 31 | If yes, who has done any of these things to physically hurt you?  1 = Current wife / partner; 2 = Other, specify  [*Please counsel and support the man to disclose this to the focal person for gender-based violence at the health facility, or the Local Council I leader (LCI)]* |  | |
| 32 | If current wife / partner, was this related to a discussion about family planning? Yes / No |  | |

| **QUESTIONS FOR VHT** | | |
| --- | --- | --- |
| Question | | Response |
| 33 | Did you do counselling at this visit about use of family planning after giving birth?: Yes/No **If No Skip to question 37** |  |
| 34 | - - Who was counselled?     - 1=Woman only 2=Woman and her partner together 3=Partner only 4=Other family member/friend |  |
| 35 | For intervention areas only; Was Drama film shown at this visit? Yes/No |  |
| 36 | For intervention areas only; Was Documentary film shown at this visit? Yes/No |  |
| 37 | Why was counselling not done during this visit? |  |
| 38 | Any other comment about this visit |  |

### **D 5. POSTPARTUM VISIT 3 (12 MONTHS)**

| **INFORMATION RECORDED AT EACH VISIT** | | |
| --- | --- | --- |
|  | Item | Response |
| 1 | VHT code | __/___/ |
| 2 | Participant ID | __/___/___/ |
| 3 | Date of visit | Day/month/year |
| 4 | Is the partner present during this meeting? Yes/No |  |

| Question | | Response |
| --- | --- | --- |
| 5a | - Is the baby still alive? Yes/No |  |
| 5b | - In case of twins; is the second baby still alive? Yes/No |  |
| 5c | - How old was the baby when s/he died? |  |
| 5d | - Do you know the possible cause of death? If yes, please specify: |  |
| 6 | - Has the baby already received measles vaccination? Yes /NO |  |
| 7 | - Have your monthly periods returned? Yes / No |  |
| 8 | Are you breast feeding the baby? Yes/No |  |
| 9 | - Have you had sexual intercourse since delivery Yes/ No |  |
| 10 | - Are you pregnant? 1=Yes 2=No 3=Not sure |  |
| 11 | Do you desire to have another child? Yes/No/Not sure  **If No or not sure, skip to qn 13** |  |
| 12 | When do you want to get pregnant? |  |
| 13 | - - Since you delivered, have you used any family planning method? Yes/No **If No, skip to qn 23** |  |
| 14 | - - Are you still using the method? Yes/No **If yes, Skip to qn 16** |  |
| 15 | - - Why have you stopped using it? **Skip to qn16** |  |
| 16 | - Did you start using family planning after our last visit? Yes/No - **If No skip to qn 19** |  |
| 17 | - If yes, when did you start? (count in months after giving birth) |  |
| 18a | Which method are you using? 1= implant, 2= IUD, 3=Sterilisation 4= Pills 5= Injectable 6= Other, specify |  |
| 18b | - - Did you need to spend any money in order to get the family planning? Yes / No |  |
| 18c | If Yes, how much did you spend? |  |
| 19 | - Have you experienced any problems / side-effects? Yes/No - **If no, skip to qn 23** |  |
| 20 | - If yes, did you go to the health facility about this? Yes/No - **If No, skip to question 23** |  |
| 21 | - - If yes, did you receive treatment? Yes/No |  |
| 22a | - - Did the treatment help? Yes/No |  |
| 22b | - - Did you need to spend any money in order to treat the side-effects? Yes / No |  |
| 22c | - - If yes, how much did you spend? |  |
| 23 | - Who made the final decision about using or not using family planning?   - 1= Woman alone, 2= Male partner alone, 3= Joint decision of the couple, 4= woman with another person apart from male partner |  |
| 24 | How do you score the various formats of information on contraception received on a scale of 10,; one being very poor and 10 being excellent | |
|  | 1. Discussion with VHT |  |
|  | 1. Discussion with health worker |  |
|  | 1. Discussion with friends and other members of the family |  |
|  | 1. Audio-visual **documentar**y films (Only for intervention areas) |  |
|  | 1. Audio-visual **drama** films (Only for intervention areas) |  |

| **PRIVATE QUESTIONS TO WOMAN** | | |
| --- | --- | --- |
| 25 | Are you using Family planning in secret without the knowledge of spouse? Yes /No **If No skip to qn 27** |  |
| 26 | If yes, Which method are you using? 1= Implant 2= IUD 3= other specify **End** |  |
| 27 | Would you like to use Family planning in secret without the knowledge of your spouse? Yes /No **If No, Skip to Q29** |  |
| 28 | Which method would you like to use? 1= Implant 2= IUD 3= other specify |  |
| 29 | Since my last visit to you, has any one hit, slapped, kicked, or done anything else to hurt you physically? Yes / No **If No, END.** |  |
| 30 | If yes, were you seriously injured (needing to go to hospital)? Yes / No  [If yes, complete serious adverse event form] |  |
| 31 | If yes, who has done any of these things to physically hurt you?  1 = Current husband / partner; 2 = Other, specify  [*Please counsel and support the woman to disclose this to the focal person for gender-based violence at the health facility, or the Local Council I leader (LCI)]* |  |
| 32 | If current husband / partner, was this related to a discussion about family planning? Yes / No |  |
| **For man only and in privacy** | | |
| 33 | Since my last visit to you, has any one hit, slapped, kicked, or done anything else to hurt you physically? Yes / No **If No, END.** |  |
| 34 | If yes, were you seriously injured (needing to go to hospital)? Yes / No  [If yes, complete serious adverse event form] |  |
| 35 | If yes, who has done any of these things to physically hurt you?  1 = Current wife / partner; 2 = Other, specify  [*Please counsel and support the man to disclose this to the focal person for gender-based violence at the health facility, or the Local Council I leader (LCI)]* |  |
| 36 | If current wife / partner, was this related to a discussion about family planning? Yes / No |  |

END
